# Cancer-associated fibroblasts facilitate breast cancer progression through exosomal circTBPL1-mediated intercellular communication

**DOI:** 10.1038/s41419-023-05986-8

**Published:** 2023-07-26

**Authors:** Fangzhou Ye, Yiran Liang, Yajie Wang, Robert Le Yang, Dan Luo, Yaming Li, Yuhan Jin, Dianwen Han, Bing Chen, Wenjing Zhao, Lijuan Wang, Xi Chen, Tingting Ma, Xiaoli Kong, Qifeng Yang

**Affiliations:** 1 Department of Breast Surgery, General Surgery, Qilu Hospital of Shandong University, 250012 Jinan, Shandong P. R. China; 2 Shandong Experimental High School, 250001 Jinan, Shandong P. R. China; 3 Pathology Tissue Bank, Qilu Hospital of Shandong University, 250012 Jinan, Shandong P. R. China; 4 Research Institute of Breast Cancer, Shandong University, 250012 Jinan, Shandong P. R. China

**Keywords:** Breast cancer, Non-coding RNAs, Cancer microenvironment

## Abstract

Breast cancer is the major common malignancy worldwide among women. Previous studies reported that cancer-associated fibroblasts (CAFs) showed pivotal roles in regulating tumor progression via exosome-mediated cellular communication. However, the detailed mechanism underlying the exosomal circRNA from CAFs in breast cancer progression remains ambiguous. Here, exosomal circRNA profiling of breast cancer-derived CAFs and normal fibroblasts (NFs) was detected by high-throughput sequencing, and upregulated circTBPL1 expression was identified in CAF exosomes. The exosomal circTBPL1 from CAFs could be transferred to breast cancer cells and promoted cell proliferation, migration, and invasion. Consistently, circTBPL1 knockdown in CAFs attenuated their tumor-promoting ability. Further exploration identified miR-653-5p as an inhibitory target of circTBPL1, and ectopic expression of miR-653-5p could partially reverse the malignant phenotypes induced by circTBPL1 overexpression in breast cancer. Additionally, TPBG was selected as a downstream target gene, and circTBPL1 could protect TPBG from miR-653-5p-mediated degradation, leading to enhanced breast cancer progression. Significantly, the accelerated tumor progression triggered by exosomal circTBPL1 from CAFs was confirmed in xenograft models. Taken together, these results revealed that exosomal circTBPL1 derived from CAFs contributed to cancer progression via miR-653-5p/TPBG pathway, indicating the potential of exosomal circTBPL1 as a biomarker and novel therapeutic target for breast cancer.

## Introduction

Breast cancer, a highly heterogeneous disease, ranks as the most prevalent malignancy and the second leading cause of cancer-related deaths in women around the world, representing a global disease burden [1]. The incidence rate of breast cancer has shown an increasing trend year by year, and late diagnosis and presence of metastasis at advanced stages remain the major causes of the high mortality [2]. The prognosis of early breast cancer patients is relatively good, however, metastatic patients are suffering a worse prognosis with only 25% 5-year survival rate [3, 4]. Despite significant improvements have been made in the diagnosis and treatment strategies of breast cancer, the prognosis remains unsatisfactory. Therefore, it is urgent and necessary to elucidate the underlying mechanism of breast cancer progression to find novel biomarkers and therapeutic targets for the disease.

Recently, more emphasis has been placed on the tumor microenvironment (TME), which consists of tumor cells, stromal and immune cells, and the surrounding factors secreted by these cellular populations [5, 6]. In many situations, tumor cells could deplete immune-related cells or educate adjacent fibroblasts to transform into activated cancer-associated fibroblasts (CAFs), contributing to tumorigenesis and tumor progression [7–9]. Increasing evidence demonstrated that CAFs, the major cellular component of the cancer stroma, could promote tumor growth, angiogenesis, and metastasis through laying down extensive extracellular matrix and releasing various pro-oncogenic signals [10, 11]. Several evidences revealed that the cooperative signaling loops between cancer cells and their surrounding CAFs could influence the therapeutic response and prognosis of cancers [12, 13], indicating the predictive and prognostic value of CAFs and their secreting factors.

Accumulating evidences revealed that exosomes, a class of extracellular vesicles with a diameter between 30 and 150 nm secreted from almost all cells [14, 15], served as critical messengers that mediate the intercellular communication in cancers[16]. Moreover, the dynamic crosstalk between CAFs and cancer cells mediated by exosomes could help to shape the tumor microenvironment [17, 18], further promoting tumor progression. Exosome cargo contains various molecules [19, 20], such as RNAs (including mRNAs, miRNAs, lncRNAs, and circRNA), proteins, and lipids, which were transferred into recipient cells and regulate their behaviors. For example, CAFs-derived miR-590-3p enhanced radioresistance through regulating CLCA4-dependent PI3K/Akt signaling pathway in colorectal cancer [21]. In addition, CAFs secreted exosomes containing LINC00659 into tumor environment, thereby facilitating colorectal cancer cell progression through miR-342-3p/ANXA2 axis [22]. Circular RNAs (circRNAs), a unique type of RNA transcripts with covalently closed loop structure, are generated from linear transcripts through back-splicing [23]. Mounting evidence indicated that circRNAs participated in the development of many human diseases, especially cancer [24, 25]. Recent studies have manifested the important role of exosome-carried circRNAs in cancer progression. For instance, exosomal circWDR62 promoted temozolomide resistance and progression of glioma through regulating miR-370-3p/MGMT axis [26]. Hypoxic exosomal circZNF91 could be transferred into normoxic pancreatic cancer cells and sponge miR-23b-3p to deprive its inhibition on expression of Sirtuin1, leading to enhance glycolysis and Gemcitabine resistance of pancreatic cancer cells [27]. However, the detailed role of circRNAs in CAF-induced cancer progression and the underlying mechanism in breast cancer have not been elucidated.

In this study, based on high-throughput sequencing and a series of experimental verifications, a novel circRNA termed circTBPL1 (hsa_circ_0077892) was identified, which was prominently enriched in CAF-derived exosomes. Subsequently, the transmission of exosomal circTBPL1 from CAFs to breast cancer cells and its malignant effects as well as regulatory axis were demonstrated using in vitro and in vivo experiments. These findings demonstrated for the first time that CAF could regulate the breast cancer progression through exosomal circRNA, and provided novel insights into the underlying molecular mechanism of how CAF-derived exosomal circTBPL1 facilitated cancer progression, indicating the possibility of circTBPL1 to serve as a potential biomarker and therapeutic target for breast cancer.

## Results

### CAF-derived exosomes exhibit upregulated expression of circTBPL1

The CAFs and NFs were isolated from breast cancer tissues and adjacent normal tissues in the study. To confirm the purity and phenotype of NFs and CAFs, the fibroblast biomarkers were evaluated using immunofluorescence and western blot, which were closely associated with the malignancy grade of tumors and metastasis [10]. NFs and CAFs were both spindle-shaped and vimentin-positive, and no significant difference in the protein expression of vimentin was identified between NFs and CAFs (Supplementary Fig. S1A, B). In addition, the expression of α-smooth muscle actin (α-SMA), fibroblast activation protein (FAP), and fibroblast-specific protein 1 (FSP1) was higher in CAFs compared with NFs (Supplementary Fig. S1A, B), further confirming the identity of the isolated cells. In order to evaluate the effect of CAFs and NFs on the malignant behaviors of cancer cells, conditioned medium (CM) from CAFs or NFs or control medium (DMEM) were used to treat breast cancer cells. The results indicated that CM from CAFs could remarkably increase the proliferation and migration abilities of breast cancer cells (Supplementary Fig. S2A–D), in consistent with previous findings [28, 29].

**Fig. 1:**
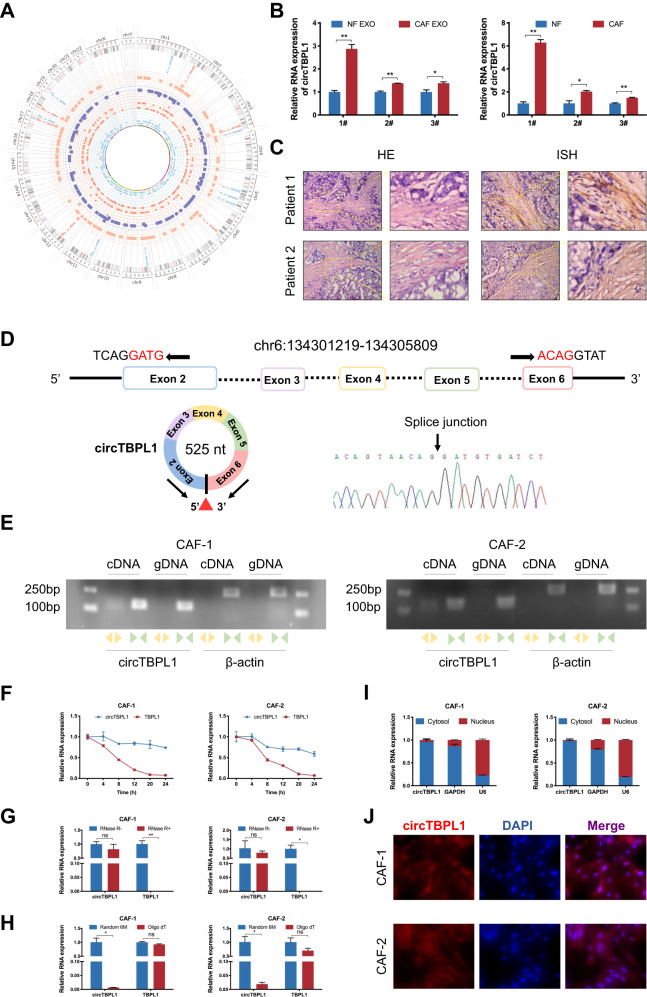
Exosomes derived from CAFs exhibit upregulated circTBPL1 expression. **A** The Circos plot shows the differentially expressed circRNAs in exosomes derived from CAFs and NFs. The outermost circle shows the chromosomal distribution of the circRNAs. The second circle indicates the circBase ID of the predominantly enriched circRNAs in exosomes derived from CAFs (orange) or NFs (blue). The third circle shows the log_2_FC of differentially expressed circRNAs (orange squares for upregulated circRNAs and purple squares for downregulated circRNAs). The fourth circle shows the number of reads the differentially expressed circRNAs matched after normalization (orange circles for CAFs and blue triangles for NFs). **B** The qRT-PCR analysis showing the circTBPL1 expression in exosomes secreted by NFs and CAFs (left) and that in the corresponding NFs and CAFs (right). **C** ISH assay was performed to detect the localization of circTBPL1 in breast cancer tissue, and HE staining was used to show the histology. **D** Schematic illustration showing the genomic location and splicing pattern of circTBPL1. The back-splice junction (red triangle) of circTBPL1was confirmed by sanger sequencing. **E** cDNA and gDNA of CAFs were amplified using convergent and divergent primers. β-actin was used as the negative control. **F** The qRT–PCR analysis was used to detect the expression of circTBPL1 and linear TBPL1 in CAFs treated with actinomycin D for indicated time. **G** The qRT–PCR analysis was performed to evaluate the expression of circTBPL1 and linear TBPL1 in CAFs treated with RNase R. **H** The expression of circTBPL1 and linear TBPL1 were evaluated by qRT-PCR using random primers or oligo dT primers. **I** The expression of circTBPL1 in the cytoplasm and nucleus of CAFs was evaluated by qRT–PCR. GAPDH and U6 were used as cytoplasmic and nuclear RNA markers, respectively. **J** FISH assay was used to detect the subcellular localization of circTBPL1 in CAFs. (ns, no significance, *P < 0.05, **P < 0.01).

Given the significant role of exosomes in the communication among cells in the tumor microenvironment, we further investigated whether the tumor-promoting role of CAFs could be mediated by exosomes in breast cancer. Using differential ultracentrifugation, exosomes were separated from CAF- and NF-derived CM. Western blot indicated positive expression of exosome marker proteins, including CD63 and CD9, and negative expression of HSP70 and GM130 in exosomes (Supplementary Fig. S3A). Moreover, the morphology of extracted exosomes was further analyzed using transmission electron microscopy (TEM), and exosomes appeared as discoid-like vesicles (Supplementary Fig. S3B). The diameter of isolated exosomes was mainly distributed between 100 and 150 nm (Supplementary Fig. S3C), in accord with the standard size of exosomes. Next, we labeled separated exosomes with fluorescent dye PKH26 and incubated them with breast cancer cells. As expected, red fluorescence signals could be observed in cancer cell after treated with exosomes, validating that PKH26-labeled exosomes were successfully internalized with breast cancer cells (Supplementary Fig. S3D). To explore the underlying mechanism contributing to the effect of CAFs, we detected the circRNA enrichment in the exosomes derived from the CM of CAFs or NFs using RNA-seq. The results indicated that 131 circRNAs were upregulated and 170 circRNAs were downregulated in the exosomes obtained from CAFs (Fig. 1A and Table S1). We focused on those enriched circRNAs derived from exosomes of CAFs, which might have significant tumor-promoting potentials and become treatment and diagnosis biomarkers. Subsequently, the top 6 upregulated circRNAs with circBase annotation were selected for further validation by qRT-PCR. Among them, the expression of circTBPL1 was increased in exosomes derived from all of 3 CAFs compared to that in paired NFs (Fig. 1B and Supplementary Fig. S3E). Consistently, the elevated expression of circTBPL1 was also confirmed in all of 3 corresponding CAFs compared to paired NFs (Fig. 1B). Therefore, circTBPL1 was selected for further functional evaluation and mechanical exploration. Significantly, in situ hybridization (ISH) assay revealed that although circTBPL1 was detected in both the cancer nest and tumor stroma, circTBPL1 was more highly expressed in tumor stroma compared to cancer nests (Fig. 1C). In addition, qRT-PCR results revealed that the circTBPL1 expression in CAFs were generally higher compared to breast cancer cells (Supplementary Fig. S3F). CircTBPL1 is generated from the back-splicing of five exons of TBPL1 gene located on human chromosome 6 with a length of 525 nt, which was preliminarily validated by sanger sequencing (Fig. 1D). Next, convergent and divergent primers was used for amplification of circTBPL1 and β-actin in CAFs by PCR. The results exhibited that the convergent primers could amplify the circTBPL1 and β-actin both in cDNA and gDNA, while only circTBPL1, rather than β-actin, could be amplified with divergent primers in cDNA (Fig. 1E), which further verified the specific circular structure of circTBPL1 in CAFs. To confirm the stability of circTBPL1, we treated CAFs with actinomycin D, an inhibitor of RNA synthesis, and observed that circTBPL1 was much more stable compared to the linear TBPL1 mRNA (Fig. 1F). In addition, circTBPL1 was more resistant to the degradation effect of RNase R, while linear TBPL1 mRNA was degraded (Fig. 1G), validating its stronger stability. Moreover, compared with the results using random primers, oligo dT primers could only amplify the linear TBPL1 mRNA rather than circTBPL1, indicating that there was no poly A tail at the end of circTBPL1 sequence and confirming the specific circular structure of circTBPL1 (Fig. 1H). For further exploration of circTBPL1, cytoplasmic/nuclear fractionation assay and FISH were performed, the results indicated that circTBPL1 was dominantly located in cytoplasm of CAFs (Fig. 1I, J). These data suggested that circTBPL1 was enriched in CAFs-derived exosomes.

### CAFs enhances the breast cancer progression through exosome-transferred circTBPL1

**Fig. 2:**
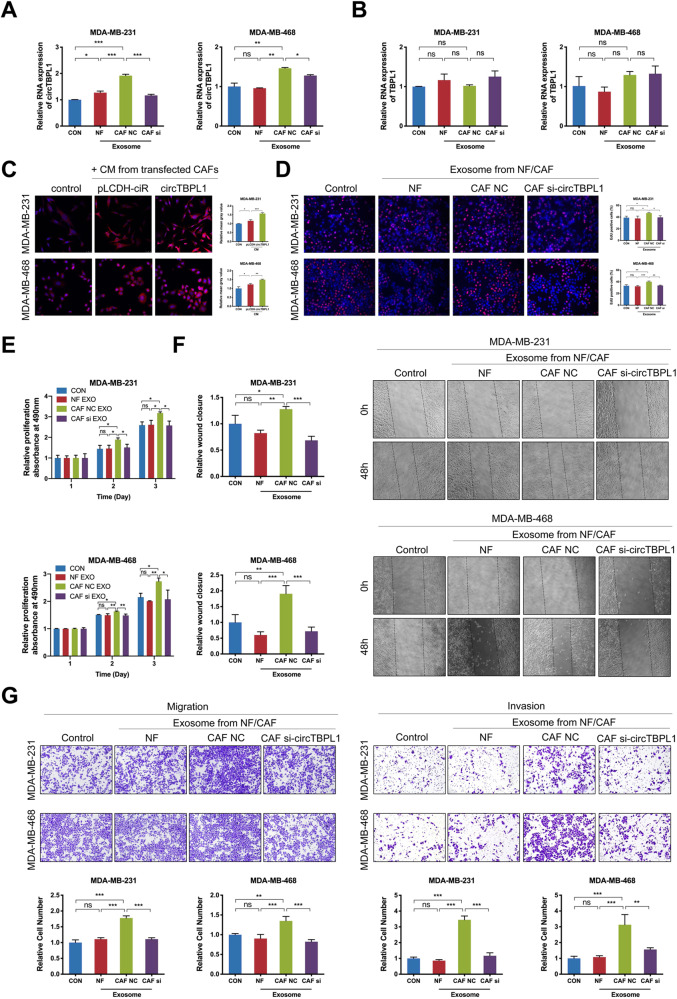
Exosomal circTBPL1 is essential for proliferation and metastasis of breast cancer cells. **A**, **B** The qRT-PCR analysis of circTBPL1 (**A**) and linear TBPL1 (**B**) expression in breast cancer cells treated with or without indicated exosomes. **C** The expression level of circTBPL1 in breast cancer cells treated with CM from CAFs was detected by FISH assay. **D**, **E** Edu (**D**) and MTT (**E**) assays showed the effect of exosomes on the proliferation of breast cancer cell. **F** Wound healing assay indicated the influence of exosomes on the migration of breast cancer cells. **G** Transwell assay showed the impact of exosomes on breast cancer cell migration and invasion. (ns, no significance, *P < 0.05, **P < 0.01, ***P < 0.001).

In order to further explore whether the biological significance of CAF on breast cancer cells was mediated by exosomal circTBPL1, we isolated exosomes from NFs and CAFs transfected with si-NC or si- circTBPL1. Then, breast cancer cells were co-cultured with collected NF or CAF exosomes. The qRT-PCR confirmed that circTBPL1 expression was significantly upregulated upon incubation with exosomes from CAFs transfected with si-NC compared to control group or NF group, and transfection with si-circTBPL1 in CAFs led to attenuated effect on circTBPL1 expression in breast cancer cells (Fig. 2A and Supplementary Fig. S3). Moreover, the expression of TBPL1 was not changed after incubation with exosomes from CAFs or NFs (Fig. 2B). In addition, treated with CMs from CAFs led to elevated expression of circTBPL1 compared to untreated control tumor cells, and circTBPL1 overexpression could further increase the circTBPL1 expression (Fig. 2C). These results indicated that the circTBPL1 could be transported by exosomes to breast cancer cells, which was mainly derived from CAFs. Functional assays of NFs-secreted exosomes and CAFs-secreted exosomes in breast cancer cells were further performed. The assays indicated that exosomes from si-NC CAFs dramatically promoted the proliferation, migration and invasion abilities of breast cancer cells (Fig. 2D–G), while exosomes from si-circTBPL1 CAFs or NFs did not exhibit obvious tumor-promoting function, confirming the critical role of circTBPL1 in cancer progression. Together, exosomal circTBPL1 secreted by CAFs could enhance the malignant behaviors of breast cancer cells.

### CircTBPL1 promotes proliferation and metastasis of breast cancer cells

**Fig. 3:**
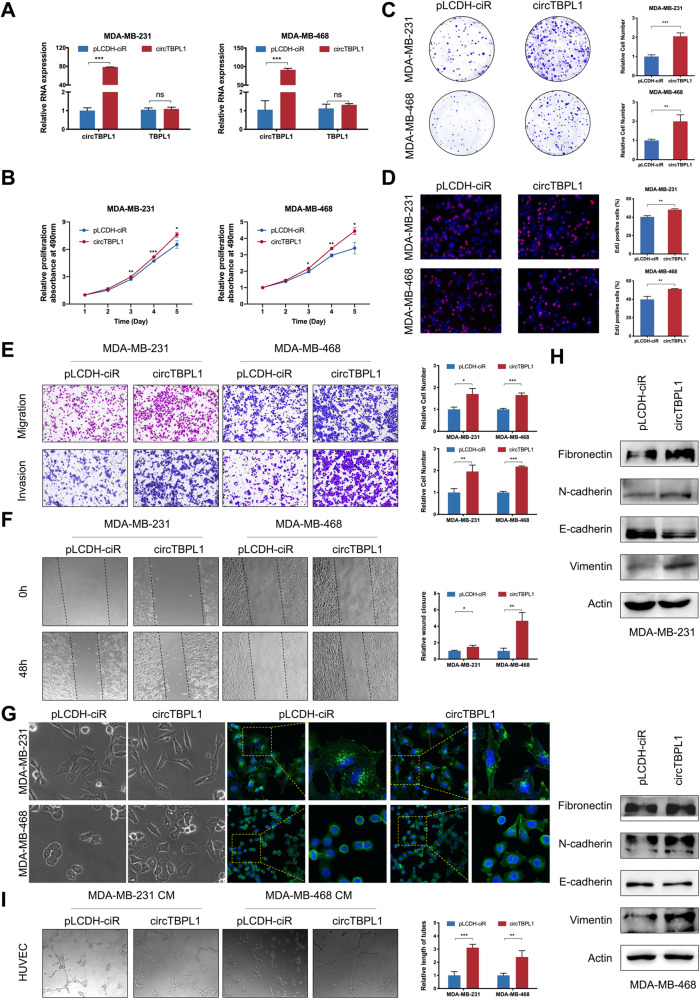
circTBPL1 overexpression promotes cell proliferation and motility of breast cancer cells. **A** The circTBPL1 overexpression efficiency and TBPL1 expression level were verified using qRT-PCR assay. **B**–**D** The proliferation of breast cancer cells after circTBPL1 overexpression was detected by MTT assay (**B**), colony formation assay (**C**), and Edu assay (**D**). **E** Transwell assay was performed to evaluate the effect of circTBPL1 overexpression on the migration and invasion abilities of breast cancer cells. **F** Wound healing assay was used to detect the cell migration after circTBPL1 overexpression in breast cancer cells. **G** Phalloidin staining was used to show the morphological change of breast cancer cells transfected with pLCDH-ciR or circTBPL1. **H** The expression of EMT-related markers was assessed by western blot. **I** Tube formation assay was performed to evaluate the angiogenesis-promoting effect of circTBPL1 overexpression in breast cancer cells. (ns, no significance, *P < 0.05, **P < 0.01, ***P < 0.001).

Based on the above results, we further investigated the tumor-associated effect of circTBPL1 on breast cancer cells. We transfected MDA-MB-231 and MDA-MB-468 cells with empty vector pLCDH-ciR and circTBPL1 respectively, and the overexpression efficiency and the unaltered expression of the host gene TBPL1 were proved using qRT-PCR (Fig. 3A). The results revealed that the proliferation rates, colony formation ability, and DNA synthesis activity of breast cancer cells were significantly increased in circTBPL1 overexpression group compared to control group (Fig. 3B–D). Meanwhile, transwell assay and wound healing assay showed that the migration and invasion abilities of breast cancer cells was markedly improved after circTBPL1 overexpression (Fig. 3E, F). Given the significance of epithelial–mesenchymal transition in tumor progression, we further evaluated the effect of circTBPL1 on EMT processes. The phalloidin staining showed that circTBPL1 overexpression led to obvious morphological changes, and the circTBPL1-overexpressed cells presented a spindlier and fibroblast-like morphology compared with the corresponding control cells (Fig. 3G). In addition, western blot assay (Fig. 3H) showed that circTBPL1 overexpression led to decreased expression of epithelial marker (E-cadherin) and elevated expression of mesenchymal markers (Fibronectin, N-cadherin, and Vimentin), further indicating the enhanced EMT process. Similar promotive roles were identified in tube formation assays, and the CM from circTPBL1 overexpressing breast cancer cells led to increased angiogenesis ability of HUVECs indicated by larger number and longer length of formed tubes (Fig. 3I). Consistently, circTBPL1 knockdown produced an inhibitory effect on the proliferation, migration, and invasion abilities of breast cancer cells (Supplementary Fig. S5). These results indicated that circTBPL1 played a critical role in breast cancer progression.

### CircTBPL1 acts as a miR-653-5p sponge in breast cancer cells

**Fig. 4:**
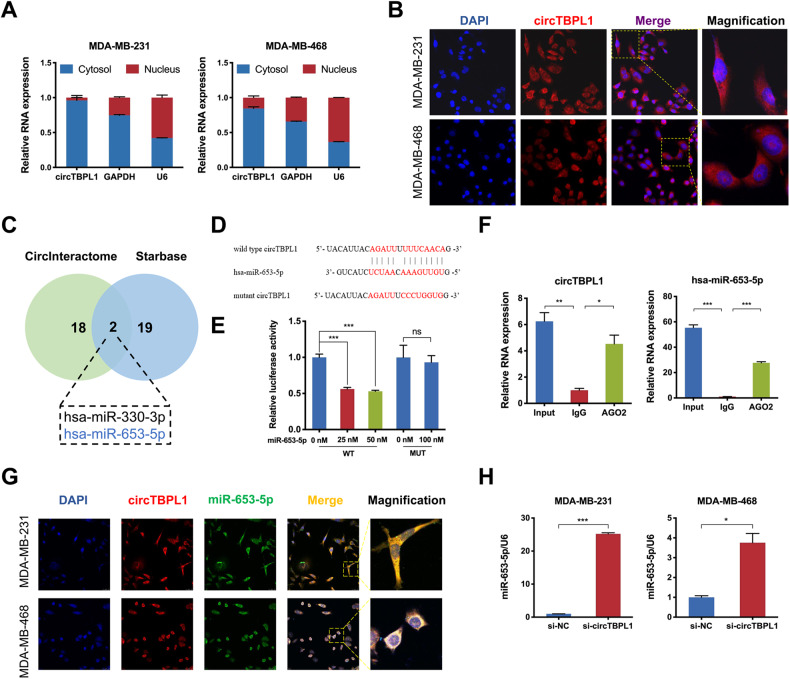
circTBPL1 serves as a miRNA sponge for miR-653-5p in breast cancer cells. **A** The expression of circTBPL1 in the cytoplasm and nucleus of breast cancer cells was evaluated by qRT–PCR. GAPDH and U6 were used as cytoplasmic and nuclear RNA markers, respectively. **B** FISH assay was used to detect the subcellular localization of circTBPL1 in breast cancer cells. **C** Venn diagram showing the potential circTBPL1-binding miRNAs, predicted by CircInteractome and Starbase databases. **D** The schematic diagram of luciferase reporter (top). The predicted circTBPL1-binding site with miR-653-5p and the corresponding mutant sequence (bottom). **E** The binding relationship between circTBPL1 and miR-653-5p was verified using the dual-luciferase reporter assay. **F** RIP assay detected the enrichment of circTBPL1 and miR-653-5p in breast cancer cells. **G** RNA-FISH showed that circTBPL1 was colocalized with miR-653-5p in breast cancer cells. **H** The miR-653-5p expression after circTBPL1 knockdown in breast cancer cells. (ns, no significance, *P < 0.05, **P < 0.01, ***P < 0.001).

Given the theoretical basis that the functions of circRNAs are closely associated with their subcellular localization [30], we further evaluated the subcellular localization of circTBPL1 in breast cancer cells. The results of subcellular fractionation and FISH assays indicated that circTBPL1 predominantly distributed in cytoplasm (Fig. 4A, B). After circRNADb database analysis, we noticed that circTBPL1 does not have open reading frame (ORF) regions (Supplementary Fig. S6A), indicating that the possibility of circTBPL1 in encoding protein is relatively low. Growing evidence has revealed that circRNAs distributed in the cytoplasm could act as miRNA sponges, binding to miRNAs and inducing altered expression of downstream molecules. To explore the potential miRNAs bound to circTBPL1, CircInteractome and Starbase databases were used, and two potential target miRNAs (miR-330-3p and miR-653-5p) were identified (Fig. 4C). Several studies have reported the enriched expression of miR-330-3p in breast cancer tissues and its tumor-promoting role [31, 32]. Consistently, our results also revealed that miR-330-3p overexpression could promote the proliferation, migration, and invasion of breast cancer cells (Supplementary Fig. S6B–F). On the contrary, miR-653-5p exhibited a universally suppressive effect on the progression of various cancers [33, 34]. Moreover, we detected that there existed a negative correlation between the expression of circTBPL1 and miR-653-5p in breast cancer cells (Supplementary Fig. S6G, H). Therefore, according to the logical relationships of ceRNAs, miR-653-5p was selected for further evaluation. One potential binding site between circTBPL1 and miR-653-5p was predicted, then we constructed two vectors with the wild type (WT) or mutant (MUT) predicted binding sites in the sequence of circTBPL1 (Fig. 4D). Dual-luciferase reporter assay indicated that only miR-653-5p mimics could evidently decrease the luciferase activity of the WT group in a dose-dependent manner, while revealed no change of luciferase activity in the MUT group, revealing the indispensable role of the predicted site for the sponging effect of circTBPL1 on miR-653-5p (Fig. 4E and Supplementary Fig. S6I). Consistently, the RIP assay found that both circTBPL1 and miR-653-5p could bind with AGO2 (Fig. 4F), a necessary component of RISC complex, which theoretically proved the feasibility of ceRNAs regulatory mechanism between circTBPL1 and miR-653-5p. Furthermore, the co-localization of circTBPL1 and miR-653-5p were detected using FISH assays (Fig. 4G). Subsequently, we inquired into the effect of circTBPL1 on miR-653-5p expression by qRT-PCR. The result uncovered that suppression of circTBPL1 increased the expression of miR-653-5p in breast cancer cells compared to the control group (Fig. 4H). These findings showed that circTBPL1 could function as a miR-653-5p sponge in breast cancer cells.

### MiR-653-5p reverses circTBPL1 overexpression-mediated effects on the malignant behaviors of breast cancer cells

**Fig. 5:**
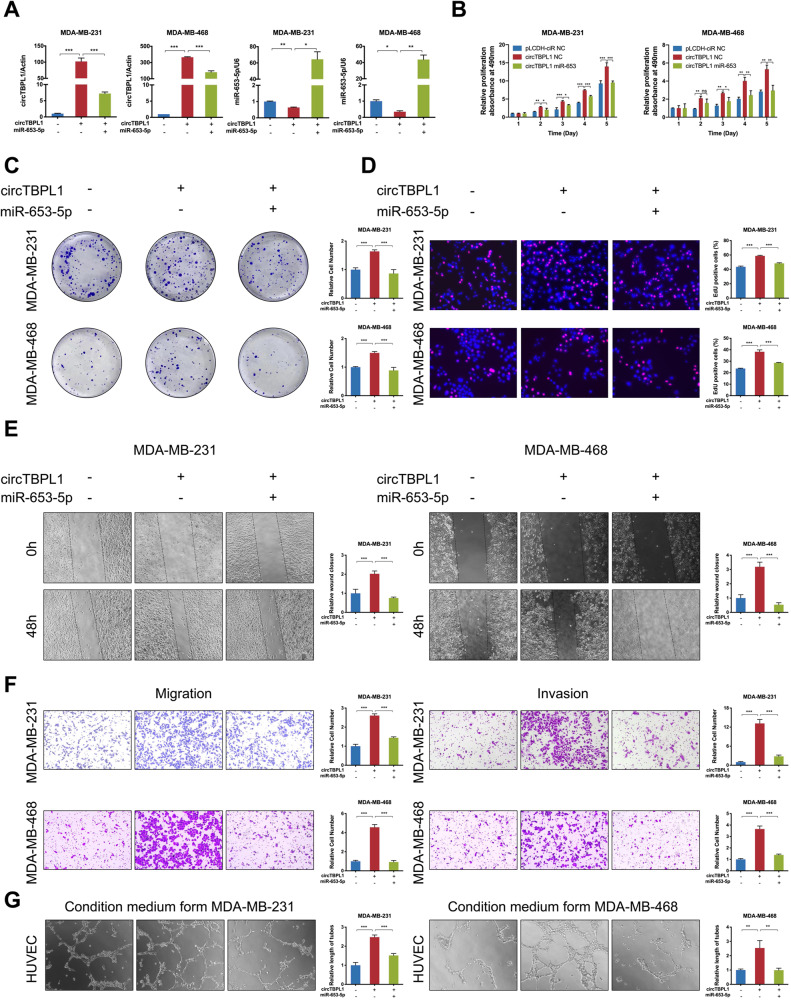
circTBPL1 promotes breast cancer progression through regulating miR-653-5p. **A** The transfection efficiency of circTBPL1 and miR-653-5p in breast cancer cells was detected using qRT-PCR. **B**–**D** MTT assay (**B**), colony formation assay (**C**), and Edu assay (**D**) were used to evaluate the proliferation ability of breast cancer cells in different transfected group. **E** Wound healing assay showed the migration and invasion abilities of breast cancer cells. **F** Transwell experiment was used to detect the metastasis ability of breast cancer cells. **G** Tube formation assay was performed to evaluate the angiogenesis-promoting effect of breast cancer cells. (ns, no significance, *P < 0.05, **P < 0.01, ***P < 0.001).

Based on the above findings, we further explored the role of miR-653-5p in breast cancer cells through a series of phenotypic experiments. The results showed that miR-653-5p significantly inhibited the proliferation rates of breast cancer cells (Supplementary Fig. S7A–D), and the migration ability, invasion ability, and angiogenesis-promoting ability appeared to be consistent with proliferation (Supplementary Fig. S7E–G). To evaluated whether miR-653-5p was involved in the malignant behaviors of breast cancer cells, rescue experiments were performed. The co-transfection of circTBPL1 overexpression vectors and miR-653-5p mimics indicated that there existed a negative regulatory relationship between circTBPL1 and miR-653-5p (Fig. 5A). MTT assay demonstrated that the proliferative rate of breast cancer cells was obviously elevated after circTBPL1 overexpression, which was partially reversed with the presence of miR-653-5p (Fig. 5B). Consistent results were identified in colony formation assay and EdU assay (Fig. 5C, D). Besides, miR-653-5p could rescue the circTBPL1-induced enhanced effect on the invasion, migration and angiogenesis-promoting abilities of breast cancer cells (Fig. 5E–G). Taken together, miR-653-5p acted as a tumor suppressor and attenuated the circTBPL1-mediated tumor-promoting functions in breast cancer.

### TPBG is directly targeted by miR-653-5p and is upregulated by circTBPL1

**Fig. 6:**
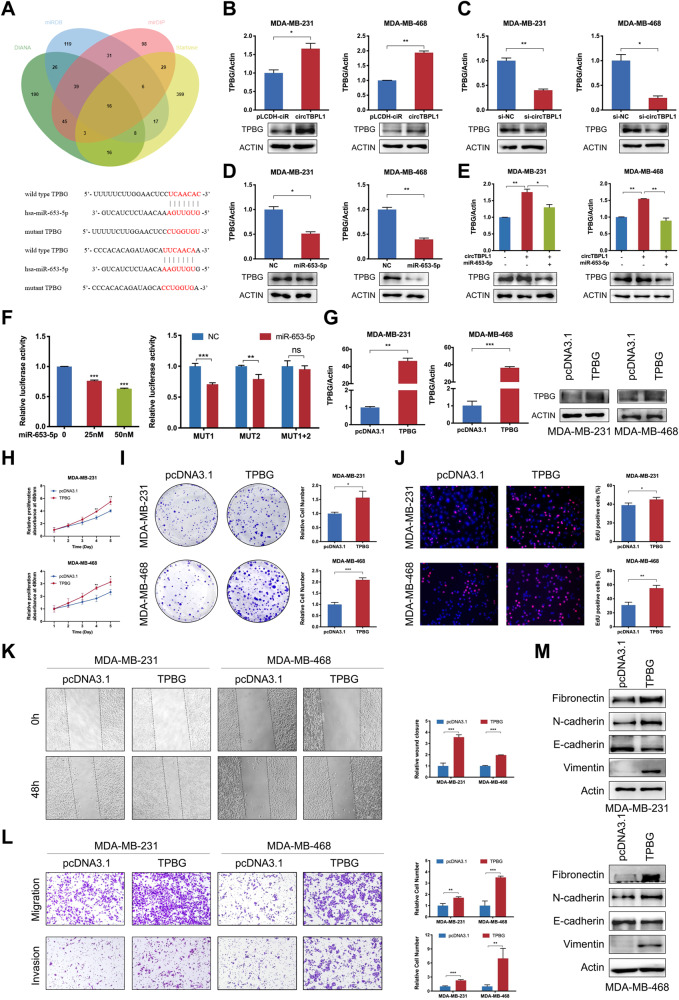
circTBPL1 sequesters miR-653-5p to promote TPBG expression. **A** Venn diagram showing the potential downstream target genes of miR-653-5p, predicted by DIANA, miRDB, mirDIP, and Starbase databases (top). The predicted miR-653-5p-binding sites with TPBG and the corresponding mutant sequence (bottom). **B**, **C** The mRNA and protein levels of TPBG after circTBPL1 overexpression (**B**) or circTBPL1 knockdown (**C**) were detected. **D** The mRNA and protein levels of TPBG after miR-653-5p overexpression were evaluated. **E** Following circTBPL1 overexpression and miR-653-5p mimics transfection, the mRNA and protein expression of TPBG was measured. **F** The binding relationship between miR-653-5p and TPBG was verified using the dual-luciferase reporter assay. **G** The overexpression efficiency of TPBG was evaluated using qRT-PCR and western blot. **H**–**J** Cellular proliferation following TPBG overexpression was assessed via MTT assay (**H**), colony formation assay (**I**), and Edu assay (**J**). **K** Wound healing assay was employed to appraise cell migration. **L** Transwell assay was implemented to analyze cell migration and invasion. **M** The expression of EMT-related markers in these cells was examined via western blot. (ns, no significance, *P < 0.05, **P < 0.01, ***P < 0.001).

Given the suppressive role of miR-653-5p and its reversed effect on the functions of circTBPL1, we wonder whether circTBPL1 could facilitate breast cancer progression through protecting tumor promoters from degradation by miR-653-5p. Four databases, including DIANA, miRDB, mirDIP, and starbase, were utilized, and 16 potential related factors were predicted after taking the intersection (Fig. 6A). Significantly, we found that miR-653-5p and circTBPL1 led to most pronounced and consistent changes in trophoblast glycoprotein (TPBG) expression (Fig. 6B–D and Supplementary Fig. S8A), which expressed positively related with circTBPL1 and simultaneously negatively related with miR-653-5p in RNA and protein levels. Moreover, TCGA database showed that the expression of TPBG was upregulated in various cancer tissues compared to normal tissues, including breast cancer (Supplementary Fig. S8B). The consistent result of TPBG protein expression was also identified in breast cancer tissues by IHC (Supplementary Fig. S8C). Therefore, TPBG was selected as the downstream of circTBPL1/miR-653-5p for further exploration. We found that the upregulated effect on TPBG by circTBPL1 overexpression could be partially abolished by miR-653-5p mimics (Fig. 6E). Besides, the exosomes from si-NC CAFs could elevated the expression level of TPBG in breast cancer cells compared with control group, NF group, and si-circTBPL1 CAF group (Supplementary Fig. S8D), which correspond to the expression alteration of circTBPL1 caused by exosomes. Next, we explored whether miR-653-5p could bind to the 3’UTR region of TPBG, as the recognized regulatory mechanism of miRNAs. There were two predicted binding sites between miR-653-5p and the 3’UTR region of TPBG (Fig. 6A). Therefore, we conducted a wild type luciferase vector and three types of mutant vector (MUT1, MUT2, MUT1 + 2) of the 3’UTR region of TPBG. The luciferase report assay indicated that miR-653-5p obviously decreased the luciferase activity of luciferase vectors containing TPBG 3’UTR in a dose-dependent manner (Fig. 6F). However, the drop of luciferase activity induced by miR-653-5p mimics was attenuated when we mutated two binding sites respectively (MUT1 or MUT2), and there exhibited no difference of luciferase activity when the two binding sites were mutant simultaneously (MUT1 + 2) (Fig. 6F). This phenomenon demonstrated that the two sites were both significantly involved in the interaction between miR-653-5p and the 3’UTR region of TPBG. Collectively, these observations indicated that TPBG served as a downstream target of miR-653-5p, and circTBPL1 could elevate TPBG expression by sponging miR-653-5p.

### CircTBPL1/miR-653-5p/TPBG axis plays a critical role in breast cancer

Considering the upregulated expression of TPBG in breast cancer tissues, we wonder whether TPBG could promote cancer progression. The overexpression efficiency of TPBG in breast cancer cells was confirmed using qRT-PCR and western blot assays (Fig. 6G). The results of MTT, colony formation, and Edu assays indicated that TPBG overexpression caused a significant increase of breast cancer cell proliferation (Fig. 6H–J). As evidenced by the wound healing and transwell assays, ectopic expression of TPBG obviously facilitated the migratory and invasive abilities of breast cancer cells (Fig. 6K, L). Moreover, western blot revealed that TPBG overexpression activated the EMT pathway in breast cancer cells (Fig. 6M). Consistently, TPBG suppression led to attenuated cell proliferation and metastasis abilities (Supplementary Fig. S9), further supporting the tumor-promoting role of TPBG in breast cancer.

**Fig. 7:**
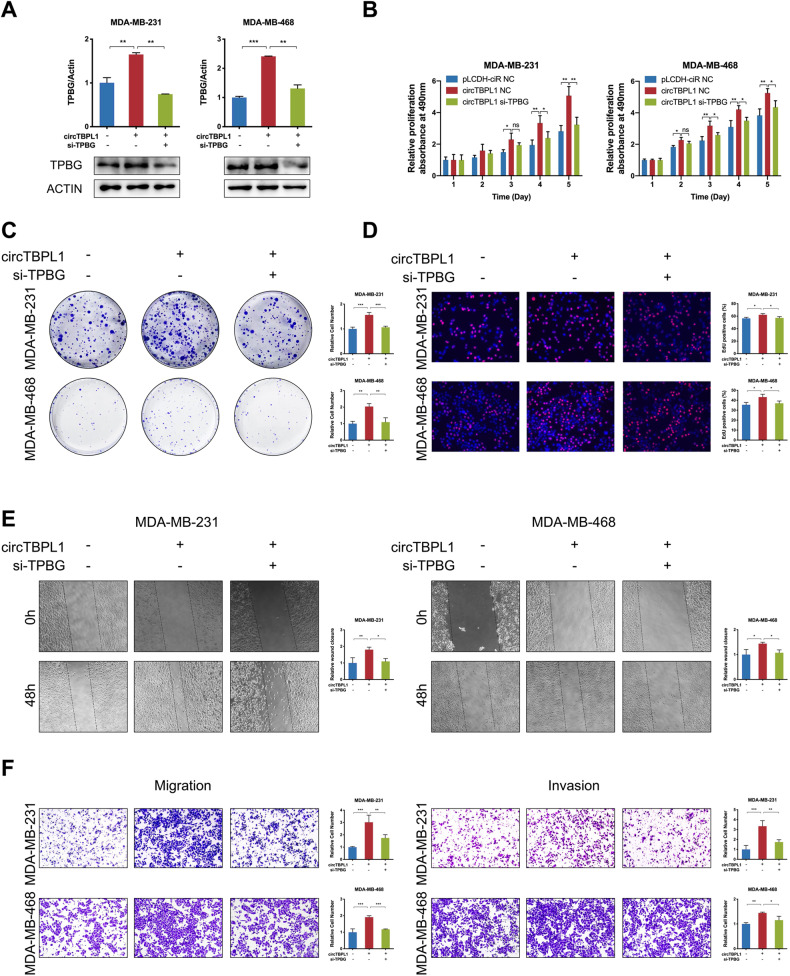
circTBPL1 promotes malignant behaviors of breast cancer cells via regulating TPBG expression. Breast cancer cells were co-transfected with circTBPL1 overexpression vectors and TPBG siRNA. **A** The RNA and protein expression of TPBG was detected using qRT-PCR and western blot. **B**–**D** The proliferation ability of breast cancer cells was evaluated by MTT assay (**B**), colony formation assay (**C**), and Edu assay (**D**). **E** Wound healing assay was performed to investigate the migration ability of breast cancer cells. **F** Transwell assay was used to evaluate the migration and invasion abilities of breast cancer cells. (ns, no significance, *P < 0.05, **P < 0.01, ***P < 0.001).

Furthermore, rescue experiments were performed to investigate the effect of TPBG on circTBPL1-mediated functions in breast cancer. The breast cancer cells were transfected with pLCDH-ciR + si-NC, circTBPL1 + si-NC, and circTBPL1+ si-TPBG, respectively. The expression of TPBG was evaluated by qRT-PCR and western blot assays (Fig. 7A). According to the MTT, colony formation, and Edu assays, knockdown of TPBG could attenuate the promoting effect of circTBPL1 on breast cancer proliferation (Fig. 7B–D). Moreover, the wound healing assay and transwell assay further confirmed that the migration and invasion abilities of cells was markedly improved after transfection with circTBPL1, and simultaneous transfection with si-TPBG could impair the effects caused by circTBPL1 overexpression (Fig. 7E, F). All these results suggested that a circTBPL1/miR-653-5p/TPBG axis existed in breast cancer, and circTBPL1 might exerts tumor-promoting effects through regulating the miR-653-5p/TPBG axis.

### Exosomal circTBPL1 promoted tumor growth and metastasis of breast cancer in vivo

**Fig. 8:**
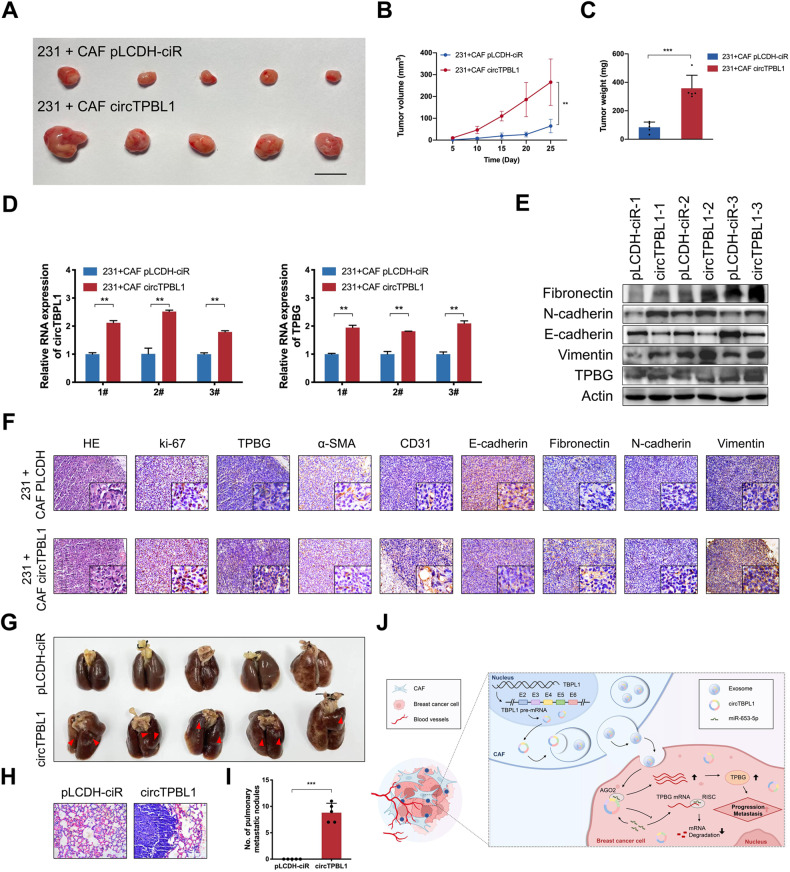
Exosomal circTBPL1 derived from CAFs regulates miR-653-5p/TPBG axis in vivo to promote tumor growth and metastasis of breast cancer. **A** Nude mice were subcutaneously implanted with MDA-MB-231 cells co-injected with CAF pLCDH-ciR or CAF circTBPL1. The images of xenograft tumors in each group. **B**, **C** Tumor volume (**B**) and weight (**C**) were measured in each group. **D**, **E** qRT-PCR (**D**) and western blot (**E**) were used to detect the expression of related factors in tumor tissues. **F** Tumor tissues from xenograft model mice were subjected to HE staining, and IHC staining was performed to evaluate the expression of the related factors in tumor tissues. **G** Representative images of the lung tissues after tail vein injection of MDA-MB-231 cells with or without circTBPL1 overexpression. **H** Representative images of HE staining for lung tissues in each group. **I** The histogram analysis of the metastatic nodules number in per lung. **J** The schematic overview illustrates the mechanistic basis for the observational study results. CAF-derived circTBPL1 is transmitted into breast cancer cells through exosomes and can therein modulate the miR-653-5p/TPBG axis to influence tumor growth and metastasis. (**P < 0.01, ***P < 0.001).

To investigate the regulatory role of circTBPL1 in vivo, we conducted subcutaneous xenograft model and lung metastasis model in nude mouse. In subcutaneous xenograft model, circTBPL1 stably overexpressed CAFs were constructed. Then MDA-MB-231 cells were subcutaneously co-injected with empty vector- or circTBPL1 stably transfected CAFs. Those tumors that were co-transplanted with CAF circTBPL1 exhibited significantly enhanced tumor growth relative to those co-injected with CAF pLCDH-ciR, which exhibited faster growth rate, larger tumor volume, and heavier tumor weight (Fig. 8A–C). Subsequent qRT-PCR assay indicated that the expression of circTPBL1 and TPBG was obviously increased in tumors from MDA-MB-231 + CAF circTBPL1 group (Fig. 8D). In addition, the protein levels of TPBG, Fibronectin, N-cadherin, and Vimentin were induced in MDA-MB-231 + CAF circTBPL1 group, while the expression of E-cadherin showed an opposite pattern (Fig. 8E), further supporting the EMT activation ability of circTBPL1 in vivo. HE staining confirmed the morphology of tumor tissues in two groups (Fig. 8F). Moreover, the IHC assay was performed to further evaluate the expression of related factors. The expression of α-SMA was confirmed in the two co-transplantation groups, whereas the mostly enhanced expression of Ki67 and TPBG in xenograft tissues was detected in MDA-MB-231 + CAF circTBPL1 group (Fig. 8F). We also detected induced expression of CD31 in MDA-MB-231 + CAF circTBPL1 group (Fig. 8F), further indicating the stronger growth ability caused by circTBPL1. We further determined the effect of circTBPL1 on breast cancer metastasis in vivo. circTBPL1 or empty vectors stably overexpressed MDA-MB-231 cell lines were constructed and injected into nude mice intravenously. The results revealed that none of the mice had pulmonary metastasis in pLCDH-ciR group, while all of the five mice had pulmonary metastasis in circTBPL1 group (Fig. 8G–I). Moreover, increased number and larger size of pulmonary metastatic foci were present in circTBPL1 group (Fig. 8G–I). Collectively, our data demonstrated that circTBPL1 derived from CAFs played a critical role in regulating tumor growth and metastasis through miR-653-5p/TPBG axis in breast cancer.

## Discussion

Breast cancer is the most frequently occurring malignancy among women, and metastasis is considered as a primary cause of cancer-related deaths. Although advancement in the diagnosis and treatment has been achieved, the clinical outcome of breast cancer patients is still not satisfactory mainly due to the existence of progression and metastasis. Therefore, elucidation of the mechanism of progression and metastasis in breast cancer to identify novel potential therapeutic targets is urgently needed. In this study, we firstly confirmed the regulating effect of exosomal circRNA derived from CAFs in breast cancer. We revealed a novel mechanism whereby CAF-derived exosomal circTBPL1 induced progression and metastasis of breast cancer, and confirmed the significant effect of circTBPL1/miR-653-5p/TPBG regulatory axis.

Recently, mounting evidence showed that TME played a key role in regulating tumor development [35], and modification of the characteristics of TME or contents in it is becoming a novel idea to improve the curative effect of cancer treatment [36, 37]. CAFs are the most plentiful and essential members of TME, which could either promote tumor progression or exhibit anti-tumor properties due to their high heterogeneity [38, 39]. Studies have revealed that CAFs could transduce some cytokines, chemokines, and growth factors, which in turn act on cells around them, including cancer cells, participating in the regulation of metastasis, angiogenesis, immunosuppression, and etc. In previous reports, CAF-derived IL32 specifically could bind to integrin β3 on the cell surface, leading to activation of p38 MAPK pathway and further facilitating invasion and metastasis of breast cancer cells [40]. The expression of IL-33 was increased in metastases-associated fibroblasts, which remodeled the immune microenvironment into a more hospitable inflammatory niche for lung metastasis of breast cancer [41]. Herein, we also confirmed that CAFs are critical regulators of tumor progression, and the CM derived from CAFs enhanced the growth and metastasis of breast cancer cells. Therefore, it can be inferred that CAFs were able to render breast cancer cells higher metastatic ability although the detailed mechanisms have not been fully clarified.

Besides those effector molecules, the tumor promoting role of exosomes-mediated intracellular communication has recently gained increasing attention, which carried and delivered complex RNAs and proteins to modulate various key processes in cancers [42]. Currently, the roles of circRNAs in the progression of breast cancer have been revealed in various studies [43]. Moreover, growing evidence recognized that circRNAs could be loaded within exosomes and transported to recipient cells to regulate the expression of target genes [44]. Therefore, we performed high-throughput sequencing to identify the specific CAFs-related circRNAs. Based on the bioinformatics analysis and qRT-PCR verification, a newly annotated and firstly reported circRNA, circTBPL1, was selected to be further investigated as the distinctly enriched exosomal circRNA derived from CAFs, which could be transferred to breast cancer cells and led to increased circTBPL1 expression. However, the biological functions and regulatory mechanisms of many other identified circRNAs in the exosomes have not been fully elucidated, and more researches are needed in the future. TATA box‑binding protein‑like protein 1 (TBPL1), the parental gene of circTBPL1, was reported to promote the progression of colorectal cancer [45, 46], however, the specific function of TBPL1 in breast cancer is unclear. In this study, functional experiments suggested that circRNA TBPL1 derived from CAFs facilitated the proliferation and metastasis of breast cancer in vitro and in vivo, serving as a critical promoter in the malignant development, which was in accordance with the role of its host gene in other cancer.

Increasing studies showed that circRNAs exerted their biological function through multiple ways [47], including regulating the expression of parental genes, serving as miRNA sponges, and acting as scaffolds to form circRNA-protein complex. To elucidate how circTBPL1 regulated breast cancer progression, we firstly investigated whether circTBPL1 influenced the expression of its parental gene-TBPL1. The results revealed the expression level of TBPL1 remained unaltered regardless of circTBPL1 overexpression or knockdown. Given that cytoplasm-located circRNAs usually contain multiple miRNA-binding sites and acting as miRNA sponges is a major regulatory way for them [48], we speculated that cytoplasmic circTBPL1 might promote breast cancer progression through this potential regulatory mechanism. The RIP assay indicated the binding between circTBPL1 and AGO2, which is essential for the biogenesis and function of miRNAs [49], supporting the potential of circTBPL1 as a miRNA sponge in breast cancer. Furthermore, based on the predictive results from two databases and literature review, miR-653-5p was selected as the circTBPL1-binding miRNA, and the interaction between circTBPL1 and miR-653-5p was confirmed by RIP and luciferase reporter assays. As reported, circRNAs might cause inactivation or degradation of their targeted miRNAs, leading to reduced miRNA levels in the cytoplasm [50, 51]. Presently, we also found that circTBPL1 could inhibit the expression of miR-653-5p. Although no statistical significance was found in the correlation analysis, the expression of miR-653-5p was negatively related with circTBPL1 in breast cancer cells, which might attribute to the limited number of cells. Interestingly, miR-653-5p was identified as a general sponging target for various non-coding RNAs, exerting a tumor-suppressing effect in many cancer types, such as prostate cancer [33], ovarian cancer [34], and melanoma [52]. Consist with the previous reports, miR-653-5p overexpression could significantly inhibit cell viability and motility, confirming the tumor-repressive role of miR-653-5p in breast cancer. Significantly, miR-653-5p overexpression could reverse the tumor-promoting effects caused by circTBPL1, which partially explained how circTBPL1 exerted its promotive role in the progression of breast cancer. Although circTBPL1 was predominantly localized in the cytoplasm, we also detected the localization of a small fraction of circTBPL1 in the nucleus. Previous studies reported that one circRNA might function through distinct mechanisms in the cytoplasm and the nucleus. Acting as miRNA sponges and the protein/circRNA interaction are significant mechanisms for the fraction of circRNAs located in the cytosol, while the nuclear circRNAs could modulate gene expression through transcription regulation, RNA splicing, and chromatin remodeling [53, 54]. For example, circPDIA4 could prevent DUSP6-mediated ERK1/2 dephosphorylation through binding with ERK1/2 to further activate MAPK signaling in cytoplasm, while the nuclear circPDIA4 could interact with the RNA-binding protein DHX9 to repress its inhibitory function on the biogenesis of various oncogenic circRNAs [55]. Moreover, circCCDC134 showed a dual tumor-promoting mechanism in cervical cancer by either recruiting p65 in nucleus or serving as a miR-503-5p sponge to regulate the expression of MYB in cytoplasm, ultimately enhancing HIF1A transcription [56]. Given the significant role and complex mechanisms of circRNAs in tumor progression, more efforts are needed to comprehensively elucidate the regulatory mechanism of circTBPL1 in the future.

Previous studies revealed that miRNAs could bind to the 3’UTR of mRNA and inhibit the expression and function of the target genes. To better identify the downstream gene regulated by miR-653-5p in breast cancer cells, four databases were analyzed and further detection assays for expression changes of the candidate genes caused by abnormal expression of miR-653-5p or circTBPL1 were performed. The results indicated that miR-653-5p had binding sites with TPBG, and miR-653-5p inhibited while circTBPL1 promoted the expression of TPBG in breast cancer cells, inferring that miR-653-5p might be a bridge for the regulation of TPBG by circTBPL1. Moreover, we also found that TPBG expression in breast cancer cells was upregulated by exosomes derived from CAFs, however, the expression of TPBG remained unchanged when treated by exosomes derived from CAFs transfected with si-circTBPL1. These findings indicated the potential roles of internalized exosomal circTBPL1 in the progression of breast cancer via regulating TPBG. Then the hypothesis that TPBG was the target of miR-653-5p in breast cancer cells was further demonstrated using luciferase reporter assays. TPBG, encoding a leucine-rich transmembrane glycoprotein that may participate in cell adhesion, has been detected to be overexpressed in a wide range of human malignant tumors. Previous studies have confirmed the significant association of TPBG with tumor malignancy and poor prognosis. Liu et al. identified TPBG as one of the nine inflammation-related genes that were used for the construction of prognosis model in lung adenocarcinoma [57]. Further experiments indicated that TPBG could serve as an effector of long non-coding RNAs LINC00342, inducing metastasis in lung adenocarcinoma through regulating miR-15b/TPBG axis [58]. Consistently, TPBG was validated to be a member of independent prognostic factors that might guide the clinical management of glioblastoma patients [59]. More impressively, it was reported that TPBG was involved in the progression of human pericyte migration as well as its angiogenic ability [60], which might be related to the enhanced angiogenesis feature of cancer. Those studies indicated that TPBG served as an oncogene in diversified tumors. However, there was no related report about the function of TPBG in breast cancer progression yet. In the present study, we found that TPBG was obviously overexpressed in breast cancer tissues, and TPBG overexpression promoted while TPBG knockdown inhibited the cell proliferative and metastatic abilities. Moreover, TPBG knockdown could reverse the aggressive phenotypes induced by circTBPL1 overexpression. In consistent with the in vitro results, exosomal circTBPL1 also promoted tumor growth in vivo by miR-653-5p/TPBG axis. Hence, these observations revealed that TPBG exhibited a tumor-promoting role, and was essential for circTBPL1-mediated breast cancer progression. However, one circRNA might participate in the tumor progression through various regulatory mechanisms and form a complex ceRNA regulatory network, and more efforts are needed to evaluate the existence and function of other circTBPL1-paricipated signal pathways in the future.

## Conclusion

Taken together, the present study demonstrated that CAF-derived exosomal circTBPL1 could facilitated the growth and progression of breast cancer, which was mediated by protecting TPBG from miR-653-5p-mediated degradation in the recipient cancer cells. Our research deepened the understanding of the complex role of CAF-derived exosomal circTBPL1 in TME and the crosstalk with cancer cells, further highlighting the potential of targeting exosomal circTBPL1 as an effective therapeutic strategy for suppressing breast cancer.

## Materials and methods

### Clinical samples and ethics statement

All the clinical samples were obtained from patients who had undergone surgery at the Qilu Hospital of Shandong University. Written informed consent was obtained from all participants. The study was approved by the Ethical Committee of Qilu Hospital of Shandong University. All of the experiments in the present study were conducted in accordance with the Declaration of Helsinki.

### Isolation and culture of patient-derived NFs and CAFs

CAFs were isolated from breast cancer tissues of patients who undergoing breast ablation and resection, and NFs were separated from the paired adjacent normal area. Tissue samples were cut up into a size of 0.5 mm × 0.5 mm × 0.5 mm and digested by collagenase (100 μg/ml) and hyaluronidase (100 μg/ml) (Stem Cell Technologies, USA) diluted in Dulbecco’s modified Eagle’s medium (DMEM; HyClone, Logan, USA) containing 10% fetal bovine serum (FBS; Sigma, St. Louis, MO, USA) at 37 °C with 70 rpm for 2 h. After centrifugation, the cells were resuspended in DMEM containing 20% FBS and then seeded into 60-mm cell culture dishes for 3-day culture at 37 °C in a humidified atmosphere containing 5% CO_2_. After stable adherence of the cells, 1×PBS was used to wash out the excess tissues and dead cells, then DMEM containing 20% FBS was added for subsequent cell culture. The paired NFs and CAFs were further characterized by the expression of specific markers.

### Immunofluorescent (IF) staining

The isolated CAFs or NFs were plated onto coverslips, washed with PBS, and then fixed in 4% paraformaldehyde for 10 min at room temperature. After permeabilization in 0.2% Triton X-100 for 15 min and blocking in 10% goat serum for 1 h, the cells were incubated with primary antibodies at 4 °C overnight. Subsequently, the cells were incubated with corresponding secondary antibodies at 37 °C for 2 h. After nuclear counterstaining with DAPI (Invitrogen), the coverslips were imaged using a fluorescent microscope (Olympus, Japan).

### Isolation and identification of exosomes

Exosomes were isolated from the conditional medium of CAFs or NFs by differential ultracentrifugation. The conditional medium was successively centrifugated at 300 × *g* and 2000 × *g* for 10 min at 4 °C respectively to remove dead cells, then the supernatant was centrifugated at 10,000 × *g* for 70 min at 4 °C to remove the cell debris and large vesicles. Next the supernatant was centrifugated at 100,000 × *g* for 70 min at 4 °C to collect the exosomes in the precipitation. Finally, the pellet was washed by 1×PBS to remove protein interference, suspended with 1×PBS, and filtered with 0.22-μm filters. Exosomes were quantified using a BCA Protein Assay Kit (Beyotime BioTech, China). For in vitro exosome treatment assays, 2 μg of exosomes (collected from approximately 2 × 10^6^ producer cells) were added to 1 × 10^5^ recipient cells in 12-well plate.

The size and morphology of exosomes were further observed by nanoparticle tracking analysis (NTA) using ZetaView PMX 110 (Particle Metrix, Meerbusch, Germany) and transmission electron microscope (FEI Tecnai G2 Spirit, Thermo Scientific, USA). To characterize the isolated exosomes, western blot analysis was performed to determine the expression of exosome markers.

### Fluorescent labeling and transfer of exosomes

PKH26 red fluorescent labeling kit (Sigma) was used to fluorescently label the exosomes according to the manufacturer’s protocol. Then the labeled exosomes were washed with PBS and centrifugated at 100,000 × *g* for 1 h to remove the residual dye. After washing twice, the labeled exosomes were incubated with breast cancer cells to determine the uptake of exosomes. After 12 h, the transfer of exosomes was observed under a fluorescence microscope (Olympus, Japan).

### Cell culture and transfection

Breast cancer cell lines (MDA-MB-231 and MDA-MB-468) were purchased from the American Type Culture Collection (Manassas, VA). The cells were conventionally cultured in DMEM containing 10% FBS, 100 mg/ml streptomycin and 100 U/ml penicillin at 37 °C in a humidified atmosphere containing 5% CO_2_.

The sequence of exons 2, 3, 4, 5, and 6 of TPBL1 with a length of 525 bp was cloned into the pLCDH-ciR vector (Invitrogen, Carlsbad, CA, USA) to construct the pLCDH-circTBPL1 plasmid. The full length of TPBG with a length of 1263 bp was cloned into pcDNA3.1 (Invitrogen, USA) to generate pcDNA3.1-TPBG construct. The related primers are listed in Table S2. The siRNA and miRNA mimics were purchased from GenePharma (Shanghai, China). The sequence of si-circTBPL1 specifically targeted the back-splice site of circTBPL1.The related sequences are listed in Table S3.

Lipofectamine 2000 reagent (Invitrogen, Carlsbad, CA, USA) was used for transient transfection. The cells transfected with pLCDH-circTBPL1 and its corresponding empty vector were screened by puromycin (2 μg/ml) for 3–4 weeks to construct stable transfected cell lines.

### RNase R treatment and actinomycin D assay

For RNase R treatment assay, total RNAs were treated with or without 3 U/µg RNase R (Epicentre, Madison, USA) at 37 °C for 30 min. For actinomycin D assay, 5 µg/ml actinomycin D (Sigma, USA) were used to treat cells for 0, 4, 8, 12, 20, and 24 h. Then qRT–PCR was used to detected circTBPL1 and TBPL1 expression levels.

### RNA extraction and quantitative real-time PCR (qRT-PCR)

Total RNA was isolated from transfected or exosome treated cells using Trizol reagent (Invitrogen, USA). In brief, 200 μl chloroform was added into 1 ml Trizol and mixed by shaking. After 10 min standing, the water phase on the top was collected after centrifugation at 12,000 × *g* 4 °C for 15 min. The same volume of isopropyl was added for RNA precipitation. 75% ethanol in DEPC water was used for washing. The RNA was finally dissolved in 20 μl DEPC water, and the concentration was detected using Nanodrop (Thermo, USA). Their complementary DNA (cDNA) was synthesized using PrimeScript reverse transcriptase (RT) reagent kit (TaKaRa, Shiga, Japan). miRNA was obtained using Mir-X miRNA First-Strand Synthesis Kit (Takara) from total RNA. qRT-PCR was carried out using SYBR Premix Ex Taq I and primers used are listed in Table S4. β-Actin and U6 were chosen as the internal control of mRNAs and miRNAs respectively.

### RNA in situ hybridization (ISH)

The Enhanced Sensitive ISH Detection kit I (BOSTER, Wuhan, China) was used to accomplish the ISH assay. The tumor tissues were sectioned into 5-µm frozen slices. The breast cancer frozen sections were fixed with 4% paraformaldehyde. Next, the slices were blocked by 30% H_2_O_2_+methanol (1:50) for 30 min. After washed three times by distilled water, the slices were digested using pepsin. Then, the slices were incubated with prehybridization solution in 37 °C for 2–4 h. A specific digoxin-labeled circTBPL1 probe was designed by GenePharma (Shanghai, China), and the sequence was as follows: AGATCACATCCTGTTACTGTGATACTTCC. The slices were hybridized with the circTBPL1 probe overnight in 4 °C. The next day, the slices were washed by gradient-diluted SSC and successively incubated with blocking reagent, biotinylation rat anti digoxin, SABC, and biotinylation peroxidase. Then, these sections were visualized using DAB.

### Collection of condition medium (CM) and cell treatment

The culture medium of transfected CAFs was changed into serum-free DMEM 48 h after transfection to collect the substance secreted by CAFs. After 24 h, the medium of CAFs was collected and subsequently centrifuged at 3000 × *g* for 15 min at 4 °C, and the supernatant was collected as CM to further treat breast cancer cells. Breast cancer cells were pre-treated with 10 or 2% FBS DMEM 1 day before CM treatment. Subsequently, related functional experiments were carried out under the CM treatment with corresponding FBS concentration.

### Western blot assay

Proteins extracted from the transfected or treated cells were separated by 10% SDS-PAGE and transferred onto 0.22-μm PVDF membranes (Millipore). Then the membranes were blocked using 5% fat-free milk for 1 h and incubated with specific primary antibodies at 4 °C overnight. After washing by TBST for three times, the blots were incubated with corresponding secondary antibodies at room temperature for 1 h and then washed another three times to remove non-specific bindings. The expression of target proteins was finally visualized using the ECL detection system (Bio-Rad, USA). β-Actin was regarded as the internal control. The primary and secondary antibodies are listed in Table S5.

### 3-(4, 5-Dimethylthiazol-2-yl)-2, 5-diphenyltetrazolium bromide (MTT) assay

The transfected or treated cells were seeded into 96-well plates with a density of 1500/well. At indicated time after incubation, 20 μl MTT (5 mg/ml) was added into each well and incubated with the cells for another 4–6 h. Then, the supernatants were removed carefully and 100 μl DMSO was added into the corresponding wells. Optical density measured at 490 nm was obtained using a microplate reader (Bio-Rad, USA) and proliferation curve was produced after calculation.

### Colony formation assay

In all, 1 × 10^3^ transfected cells were seeded into 60-mm cell culture dishes. After 2–3 weeks, the cells were washed with 1×PBS for three times, fixed by methanol for 15 min and stained with 0.5% crystal violet solution for another 15 min at room temperature. The colonies were imaged and counted.

### EdU incorporation assay

The EdU incorporation assay kit (RiboBio, China) was used for evaluation of cell proliferation. In all, 1 × 10^4^ transfected cells were seeded into 96-well plates 1 day in advance. Also, 4% paraformaldehyde was used for cell fixation and 0.5% Triton-X 100 was used to increase the permeability of cell membrane.The cells were stained with Apollo dye for 1 h and the nucleus were stained with Hoechst for 30 min in dark place. Images were obtained using the fluorescence microscope (Olympus, Japan).

### Wound healing assay

Transfected or treated cells were seeded into 24-well plates. After the density of cell reached 90%, a pipette tip (10 μl) was used to produce a wound on the monolayer of cells. Then the remained cells were washed using 1×PBS for three times and cultured in serum-free medium for 48 h. Images of the healing status were captured at 24 and 48 h, respectively, by an Olympus microscope.

### Transwell migration and invasion assay

Cell medium containing 20% FBS were added into the lower insert of the transwell chamber (pore size 8 μm; Costar Corporation, USA) and 6–8 × 10^4^ transfected or treated cells resuspended in 200 μl serum-free DMEM were added into the upper insert. Matrigel (BD Biosciences, USA) was added in advance for the detection of invasion ability. After being incubated for 24–48 h, the cells were fixed by methanol for 15 min and stained with 0.5% violet for 20 min at room temperature. The cells successfully went through the transwell membrane were pictured using a light microscope and counted for statistics.

### Fluorescence in situ hybridization (FISH)

The FISH assay was carried out to detect the location of circTBPL1 using a FISH kit (GenePharma, Shanghai, China) according to the manufacturer’s protocol. Briefly, after fixation and permeabilization, the cells were hybridized with Cy3-labeled circTBPL1 and FAM-labeled miR-653-5p probes (GenePharma, Shanghai, China) at 4 °C overnight. The cell nuclei were stained by DAPI (Sigma-Aldrich, USA). All images were visualized and obtained under a confocal microscope (Leica TCS SP8; Wetzlar, Germany). The Mean Gray Value of each image was calculated by Integrated Density / corresponding area, which were measured by Image J.

### Subcellular fractionation

The PARIS Kit (Invitrogen) was used for nuclear and cytoplasmic separation according to the manufacturer’s protocol.

### Dual-luciferase reporter gene assay

The full length of circTBPL1 and wild type cDNA fragments with predicted miRNA binding site of TBPL1 3’-UTR were amplified by PCR. The Mutated fragments were acquired using overlap extension PCR. Then, the wild-type circTBPL1 and the 3’UTR region of TPBG as well as their mutant sequences were recombined into pmirGLO vectors (Invitrogen, USA) respectively for further analysis. HEK293T cells were co-transfected with WT vectors or MUT vectors and miRNA mimics or their control mimics using Lipofectamine 2000 (Invitrogen, USA). After 48 h of incubation, the luciferase activity was detected using a dual-luciferase reporter assay kit (Promega, USA), and firefly luciferase activity was normalized with Renilla luciferase activity.

### RNA immunoprecipitation (RIP) assay

The magnetic RIP RNA-binding protein immunoprecipitation kit (Millipore, USA) was used for RIP assay according to the manufacturer’s instructions. Briefly, cells were harvested and lysed using RIP lysis buffer, then incubated with magnetic beads conjugated with primary antibodies against IgG (Millipore, USA) or AGO2 at 4 °C overnight. The RNA binding to beads were washed and purified using RNA extraction reagent. The qRT-PCR assay was performed to detect the relative enrichment abundance of circTBPL1 and miRNAs.

### In vivo tumorigenesis and metastasis assay

We construct CAFs stably expressing circTBPL1 using puromycin screening after lentivirus infection. In all, 5 × 10^6^ of breast cancer cells (MDA-MB-231) and CAFs/circTBPL1 or CAFs/pLCDH-ciR were mixed at the ratio of 1:1 in 200 μl PBS and subcutaneously injected together into female BALB/c nude mice (aged 4–6 weeks). The long and short diameters were measured every 5 days to monitor the growth rate and draw a growth curve. The tumor volume (TV) was calculated as followed: TV (mm^3^) = (length × width^2^)/2. The mice were sacrificed after 25 days and the tumors were excised and weighed. H&E staining was used to confirm the morphology of tumor tissues. For analysis of metastasis, MDA-MB-231 stably expressing circTBPL1 were constructed. Also, 5 × 10^5^ MDA-MB-231 with or without circTBPL1 overexpression were injected into the tail veins of female BALB/c nude mice (aged 4–6 weeks). After 40 days, the lungs of all the mice were collected for nodule statistics and H&E staining.

### Immunohistochemistry (IHC)

Tumor tissue samples were embedded in paraffin and sectioned to 4 μm before use. Then, the slides were deparaffinized and rehydrated. After removing the endogenous peroxidase using 3% hydrogen peroxide and antigen retrieval in a microwave, the sections were blocked with 5% BSA and then incubated with the primary antibodies overnight at 4 °C. Then the sections were incubated with horseradish peroxidase (HRP)-conjugated secondary antibodies for 2 h at room temperature. The locations of antigens were marked using DAB solution, followed by counterstaining with hematoxylin. Images were obtained under a light microscopy (Olympus). The expression level was quantified by H-score, which was determined by multiplying the score for staining intensity and the score for positive area. The intensity of staining was evaluated as follows: 0, negative; 1, weak; 2, moderate; 3, strong. The proportion of tumor cells with positive staining was corresponding to the scores as follows: 0, <5%; 1, 5–25%; 2, 26–50%; 3, 51–75%; 4, 76–100%. The primary antibodies used were listed in Table S5.

### Statistical analysis

Data are expressed as the mean ± standard deviation (SD), and statistical analysis was performed using GraphPad Prism 8 (San Diego, California, USA). Student’s *t* test was used to analyze the differences between two groups. One-way ANOVA was applied for multiple comparisons. The Pearson’s correlation coefficient analysis was performed to analyze the correlations. *P* value < 0.05 was considered statistically significant.

## Supplementary information

Supplementary Figure-1

Supplementary Figure-2

Supplementary Figure-3

Supplementary Figure-4

Supplementary Figure-5

Supplementary Figure-6

Supplementary Figure-7

Supplementary Figure-8

Supplementary Figure-9

Supplementary Figure-10

Supplementary Figure Legends

Table S1

Table S2-S5

Original Data File

aj-checklist

## Data Availability

All data are available from the corresponding author upon reasonable request.
