# Supplementary material for: Cancer-associated fibroblasts facilitate breast cancer progression through exosomal circTBPL1-mediated intercellular communication
Source: Cell Death Dis. 2023 Jul 26;14(7):471. doi: 10.1038/s41419-023-05986-8 (PMC10372047; doi:10.1038/s41419-023-05986-8)
Supplement: Supplementary file 11 — Supplementary Figure Legends [file 41419_2023_5986_MOESM11_ESM.docx]

**Supplementary Figure Legends**

**Supplementary Fig. S1 The characteristics of CAFs and NFs.** (A) The expression of α-SMA, FAP, FSP-1, and vimentin in CAFs and NFs was assessed by immunofluorescent staining. (B) The expression of α-SMA, FAP, FSP-1, and vimentin in CAFs and NFs was measured via western blotting.

**Supplementary Fig. S2 The functions of CM collected from CAFs and NFs.** (A) The Edu assay indicated that CM collected from CAFs promoted the proliferation of breast cancer cells in 10% FBS culture condition. (B) Transwell assay revealed the increased migration and invasion abilities of breast cancer cells when treated with CM collected from CAFs in 10% FBS culture condition. (C) The Edu assay indicated that CM collected from CAFs promoted the proliferation of breast cancer cells in 2% FBS culture condition. (D) Transwell assay revealed CM collected from CAFs increased migration and invasion abilities of breast cancer cells in 2% FBS culture condition. (ns, no significance, *P < 0.05, **P < 0.01, ***P < 0.001)

**Supplementary Fig. S3 The characteristics and functions of isolated exosomes.** (A) GM130, HSP70, Calnexin, CD9, and CD63 levels in cell lysis and exosomes derived from CAFs or NFs were evaluated by western blotting. (B) The exosomes derived from CAFs or NFs were analyzed via transmission electron microscopy (TEM). scale bar, 200 nm. (C) The exosomes isolated from CAFs or NFs were analyzed via nanoparticle tracking analysis. (D) The images showing PKH26-labelled exosomal uptake by breast cancer cells. (E) The expression levels of upregulated circRNAs were evaluated by qRT-PCR. (F) The expression level of circTBPL1 in cancer cells and CAFs was assessed by qRT-PCR. (ns, no significance, *P < 0.05, **P < 0.01)

**Supplementary Fig. S4 The expression of circTBPL1 in CAFs and breast cancer cells.** (A) Schematic illustration and sequence of si-circTBPL1 specifically targeting circTBPL1 back-splice site. (B) The knockdown efficiency of circTBPL1 and TBPL1 expression level in CAFs were detected by qRT-PCR.

**Supplementary Fig. S5 circTBPL1 knockdown inhibits proliferation, migration, and invasion of breast cancer cells.** (A) Levels of circTBPL1 and TBPL1 were assessed in MDA-MB-231 and MDA-MB-468 cells following si-NC or si-circTBPL1 transfection. (B-D) The cell proliferation of breast cancer cells after circTBPL1 knockdown was measured using MTT assay (B), colony formation assay (C), and Edu assay (D). (E) Wound healing assay was used to detect the effect of circTBPL1 knockdown on cell migration in breast cancer cells. (F) Transwell assay was performed to assess the migration and invasion abilities of breast cancer cells with or without circTBPL1 knockdown. (G) The expression of EMT-related markers was detected by western blot. (ns, no significance, *P < 0.05, **P < 0.01, ***P < 0.001)

**Supplementary Fig. S6 miR-653-5p, rather than miR-330-3p, was selected as the miRNA sponge of circTBP1 in breast cancer.** (A) No protein coding ability of circTBPL1 was predicted. (B) The overexpression efficiency of miR-330-3p mimics in breast cancer cells. (C-D) The MTT assay (C) and Edu assay (D) suggested that miR-330-3p overexpression promoted cell proliferation. (E) miR-330-3p overexpression enhanced cell migration based on wound healing assay. (F) Transwell assays indicated miR-330-3p overexpression enhanced migration and invasion of breast cancer cells. (G) Levels of circTBPL1 and miR-653-5p was assessed in normal breast cells and breast cancer cells. (H) The association between circTBPL1 expression and miR-653-5p expression was evaluated by Pearson’s correlation coefficient analysis. (I) Dual-luciferase reporter assay showed no binding relationship between miR-330-3p and circTBPL1. (*P < 0.05, **P < 0.01, ***P < 0.001)

**Supplementary Fig. S7 miR-653-5p overexpression inhibits the proliferation and motility of breast cancer cells.** (A) The overexpression efficiency of miR-653-5p mimics in breast cancer cells. (B-D) The MTT assay (B), colony formation assay (C), and Edu assay (D) suggested that miR-653-5p overexpression inhibited cell proliferation. (E) miR-653-5p overexpression inhibited cell migration based on wound healing assay. (F) Transwell assays indicated miR-653-5p overexpression inhibited migration and invasion of breast cancer cells. (G) Tube lengths of HUVEC cells treated with CM from breast cancer cells with or without miR-653-5p overexpression were measured by tube formation assay. (*P < 0.05, **P < 0.01, ***P < 0.001)

**Supplementary Fig. S8 TPBG is regulated by circTBPL1/miR-653-5p and upregulated in breast cancer tissues.** (A) qRT–PCR analysis of screened downstream target genes of circTBPL1 and miR-653-5p. (B) The RNA expression of TPBG in various cancer tissues and corresponding normal tissues based on TCGA database. (C) The protein expression of TPBG in breast cancer tissues and normal tissues according to The Human Protein Atlas database. (D) The qRT-PCR analysis of TPBG expression in breast cancer cells treated with or without indicated exosomes. (ns, no significance, *P < 0.05, **P < 0.01, ***P < 0.001)

**Supplementary Fig. S9 TPBG knockdown inhibited proliferation, migration, and invasion of breast cancer cells.** (A) The efficiency of TPBG knockdown was validated by qRT-PCR and western blot. (B-D) MTT assay (B), colony formation assay (C), and Edu assay (D) of si-NC and si-circTBPL1 breast cancer cells. (E) Wound healing analysis of si-NC and si-circTBPL1 breast cancer cells. (F) Transwell assay in breast cancer cells after si-NC or si-circTBPL1 transfection. (*P < 0.05, **P < 0.01, ***P < 0.001)

**Supplementary Fig. S10 Exosomal circTBPL1 derived from CAFs promote tumor growth and metastasis in vivo.** H-score was used for quantitative analysis on the related factors. (ns, no significance, *P < 0.05, **P < 0.01, ***P < 0.001)
