# Supplementary material for: Cancer-associated fibroblasts facilitate breast cancer progression through exosomal circTBPL1-mediated intercellular communication
Source: Cell Death Dis. 2023 Jul 26;14(7):471. doi: 10.1038/s41419-023-05986-8 (PMC10372047; doi:10.1038/s41419-023-05986-8)
Supplement: Supplementary file 13 — Table S2-S5 [file 41419_2023_5986_MOESM13_ESM.docx]

**Table S2. Primers used for vector construction.**

| **Name** | **Forward （5’-3’）** | **Reverse （5’-3’）** |
| --- | --- | --- |
| pLCDH-circTBPL1 | CGGAATTCGATGTGATCTTCGTGGTGG | CGGGATCCCTGTTACTGTGATACTTCCT |
| pcDNA3.1-TPBG | CGGGATCCATGCCTGGGGGGTGCTCC | CGGAATTCTCAGACATCCGAGTTAGAA |

**Table S3. SiRNA used for transfection.**

| **Name** | **sense （5’-3’）** | **antisense （5’-3’）** |
| --- | --- | --- |
| si-circTBPL1 | AACAGGAUGUGAUCUUCGU | ACGAAGAUCACAUCCUGUU |
| si-TPBG | GGCACCUGGACUUAAGUAA | UUACUUAAGUCCAGGUGCC |
| miR-653-5p | GUGUUGAAACAAUCUCUACUG | GUAGAGAUUGUUUCAACACUU |
| NC | UUCUCCGAACGUGUCACGU | ACGUGACACGUUCGGAGAA |

**Table S4. Primers used for qRT-PCR.**

| **Gene** | **Forward （5’-3’）** | **Reverse （5’-3’）** |
| --- | --- | --- |
| circTBPL1 | TCATCCTGCTGTGTGCTATCG | CTGCATCCATTGGGGTGGTT |
| TPBG | CCTCGGCATCCTCCTTCT | GGGCACCTCGGTCAGATT |
| TBPL1 | AAGCAAGGAAGACGGAGTC | CGAAGGAGAAGCTGGAGG |
| PBX1 | CTGATGCGGCTGGACAAC | GCTCTGTAATCTGAATGCTCC |
| RSF1 | GGGAAGGAGAAGATGAGGTG | CGTGTCCGAGAACCAGTCC |
| EIF1AX | GACTACCAGGATAACAAAGC | CCAGGACCAAATGTATCAGT |
| NFIA | TACAGGACCCAGAGCAAG | AGCATCAGGGCAGACAAG |
| RUFY2 | TGGAACATTGCCTGAAAC | GGTAGATCCCGGACACTA |
| SUZ12 | CCATGCAGGAAATGGAAGAATGTC | CTGTCCAACGAAGAGTGAACTGC |
| TRAM2 | TCCTCTTCCACACGGCTAGACT | ACGGCAAGGGTGAGGATGAAGA |
| TOP2B | GGTCAGTTTGGAACTCGGCTTC | AGGAGGTTGTCATCCACAGCAG |
| Actin | CACTGTGCCCATCTACGAG | AATGTCACGCACGATTTCC |
| miR-653-5p | GTGTTGAAACAATCTCTACTG | CAGTGCGTGTCGTGGAGT |
| U6 | CTCGCTTCGGCAGCACA | AACGCTTCACGAATTTGCGT |

**Table S5. Antibodies used in the experiments.**

| **Antigen** | **Supplier** | **Catalog #** | **Application** |
| --- | --- | --- | --- |
| Actin | Proteintech | 60008-1-Ig | IB (1:2000) |
| Fibronectin | Proteintech | 15613-1-AP | IB (1:2000) IHC(1:300) |
| N-cadherin | Cell Signaling Technology | 13116 | IB (1:1000) IHC(1:100) |
| E-cadherin | Cell Signaling Technology | 14472 | IB (1:1000) IHC(1:200) |
| Vimentin | Cell Signaling Technology | 5741 | IB (1:1000) IF(1:200) IHC(1:200) |
| TPBG | Proteintech | 29394-1-AP | IB (1:1000) IHC(1:200) |
| Ki67 | Cell Signaling Technology | 9449 | IHC(1:800) |
| CD31 | Proteintech | 11265-1-AP | IHC(1:800) |
| α-SMA | Cell Signaling Technology | 19245 | IB (1:1000) IF(1:200) IHC(1:400) |
| FAP | Cell Signaling Technology | 66562 | IB (1:1000) IF(1:200) |
| FSP-1 | Proteintech | 16105-1-AP | IB (1:1000) IF(1:200) |
| GM130 | Cell Signaling Technology | 12480 | IB (1:1000) |
| HSP70 | Cell Signaling Technology | 4873 | IB (1:1000) |
| Calnexin | Cell Signaling Technology | 2679 | IB (1:1000) |
| CD63 | Proteintech | 25682-1-AP | IB (1:500) |
| CD9 | Cell Signaling Technology | 98327 | IB (1:1000) |
