# Supplementary material for: Cancer-associated fibroblasts facilitate breast cancer progression through exosomal circTBPL1-mediated intercellular communication
Source: Cell Death Dis. 2023 Jul 26;14(7):471. doi: 10.1038/s41419-023-05986-8 (PMC10372047; doi:10.1038/s41419-023-05986-8)

Fig. 3G

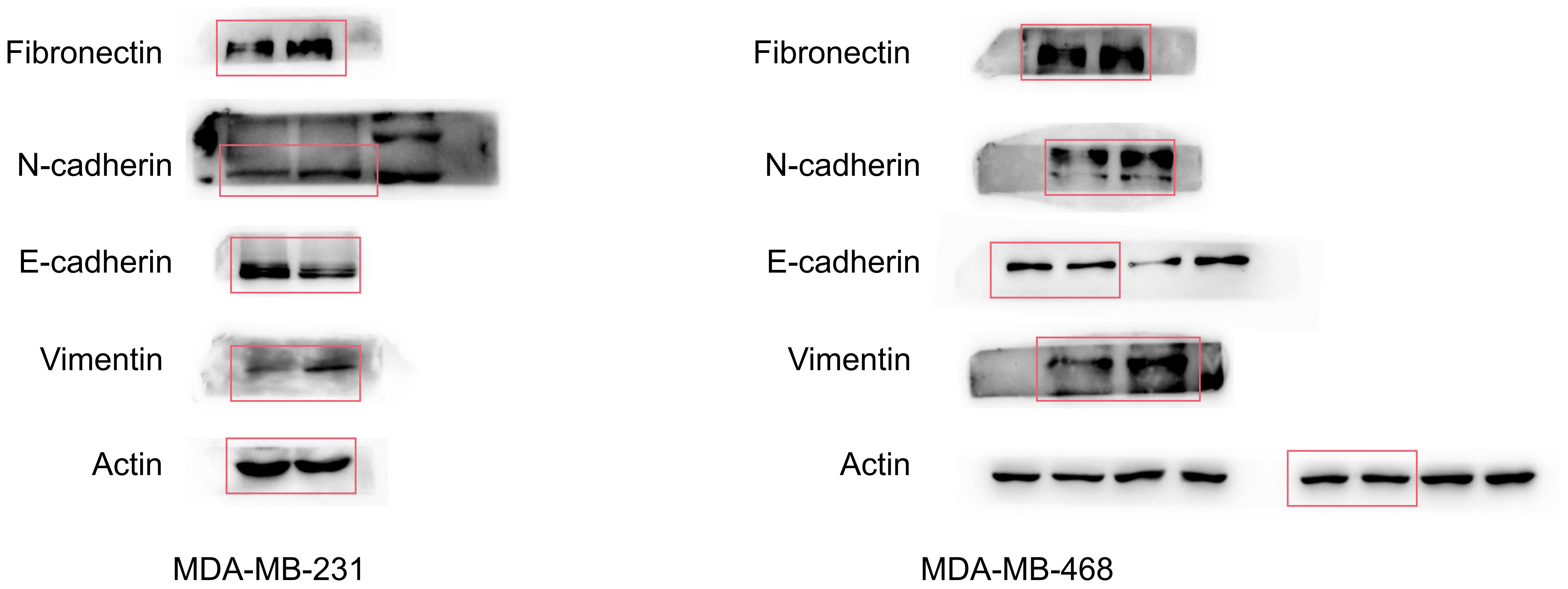
Fibronectin
N-cadherin
E-cadherin
Vimentin
Actin
MDA-MB-231
Fibronectin
N-cadherin
E-cadherin
Vimentin
Actin
MDA-MB-468

Fig. 6B

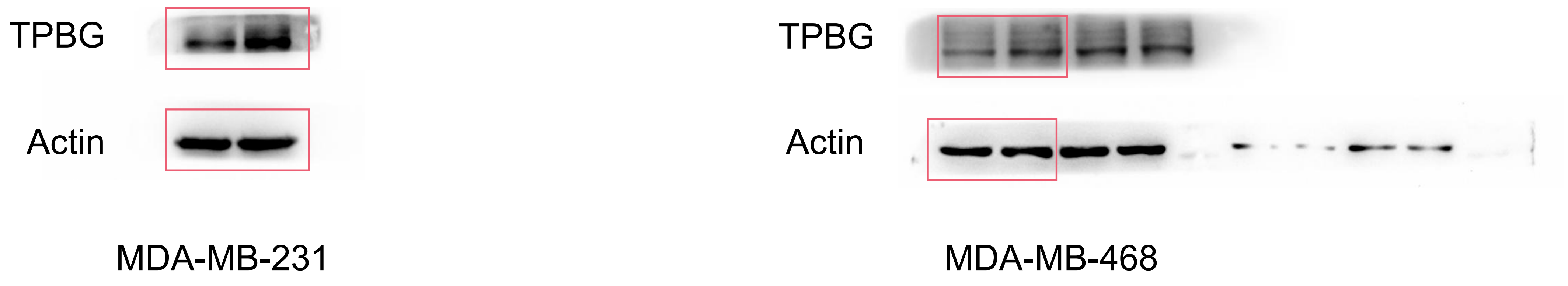
TPBG
Actin
MDA-MB-231
TPBG
Actin
MDA-MB-468

Fig. 6C

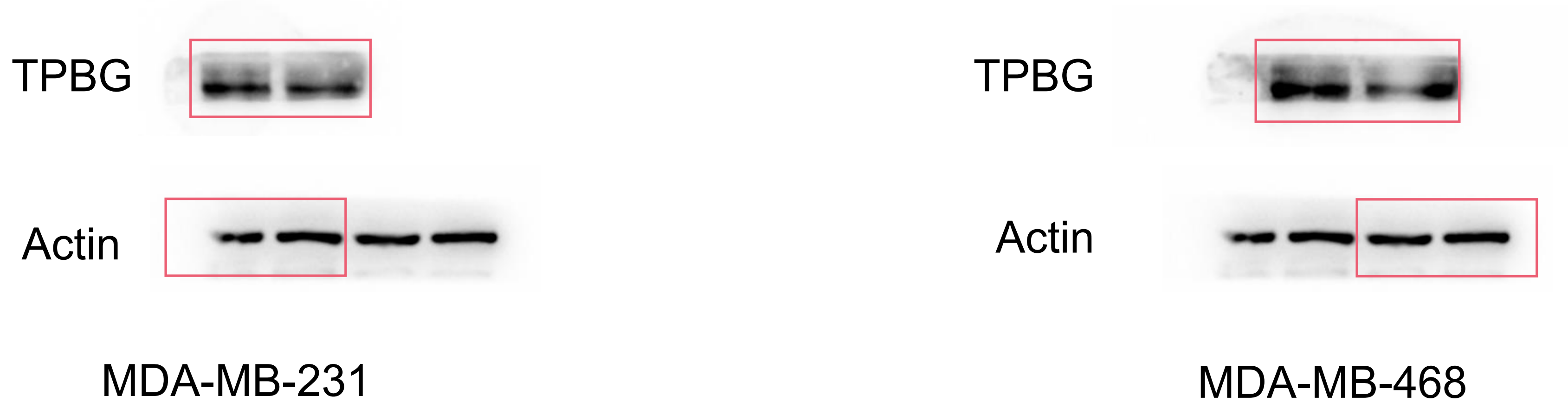
TPBG
Actin
MDA-MB-231
TPBG
Actin
MDA-MB-468

Fig. 6D

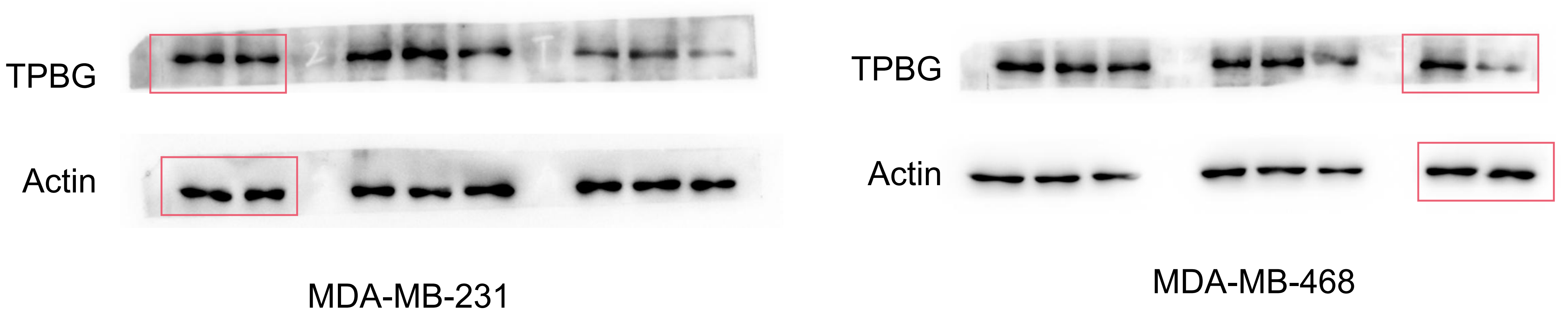
TPBG
Actin
MDA-MB-231
TPBG
Actin
MDA-MB-468

Fig. 6E

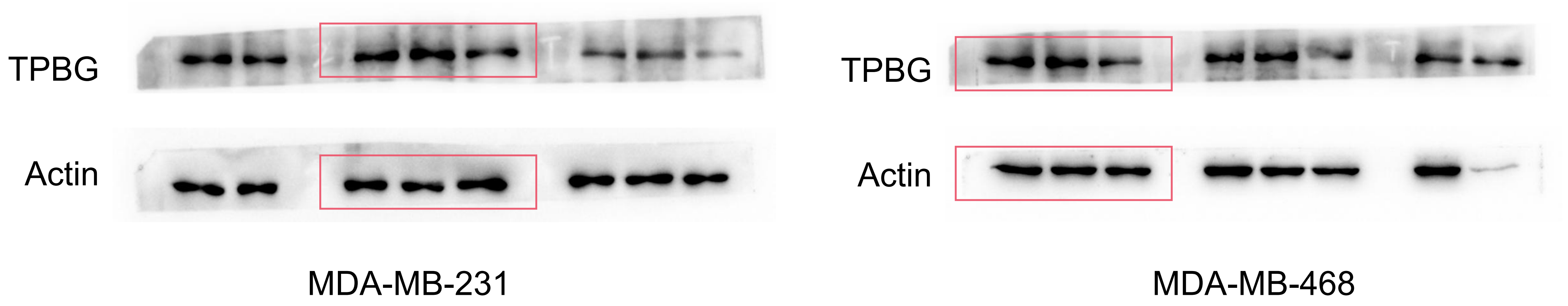
TPBG
Actin
MDA-MB-231
TPBG
Actin
MDA-MB-468

Fig. 6G

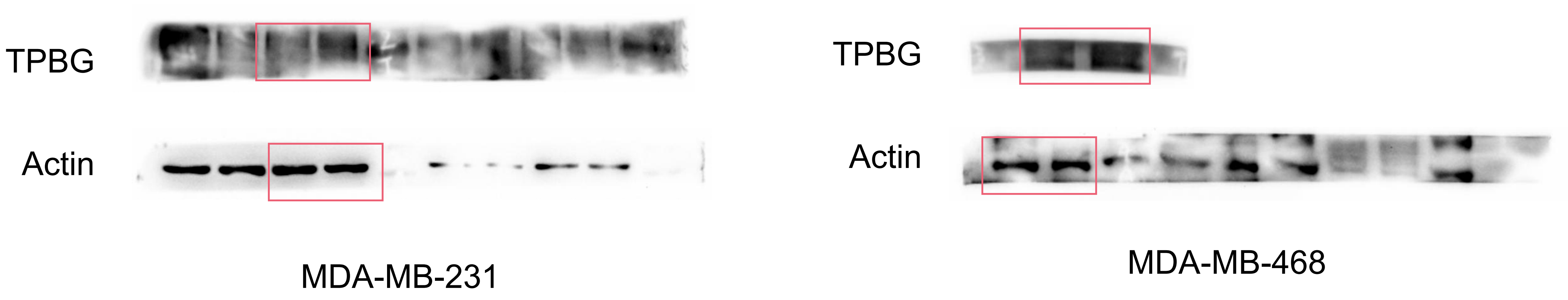

Fig. 6M

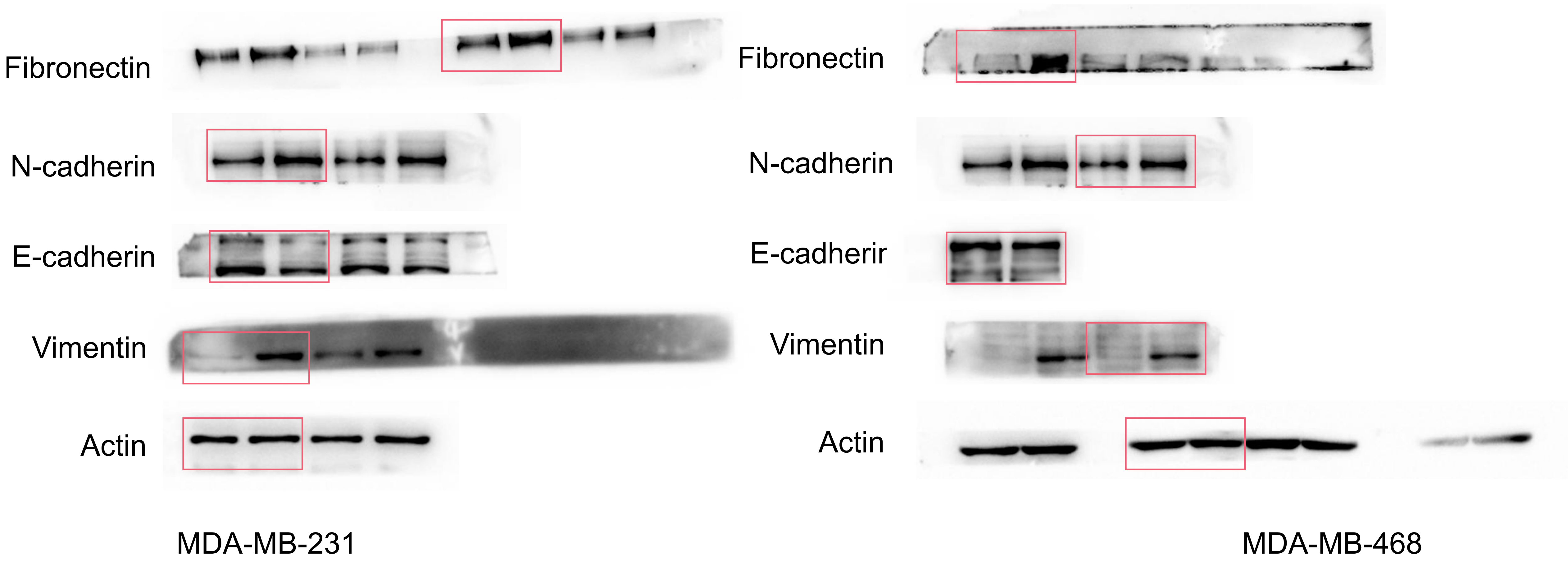
Fibronectin
N-cadherin
E-cadherin
Vimentin
Actin
MDA-MB-231
Fibronectin
N-cadherin
E-cadherir
Vimentin
Actin
MDA-MB-468

Fig. 7A

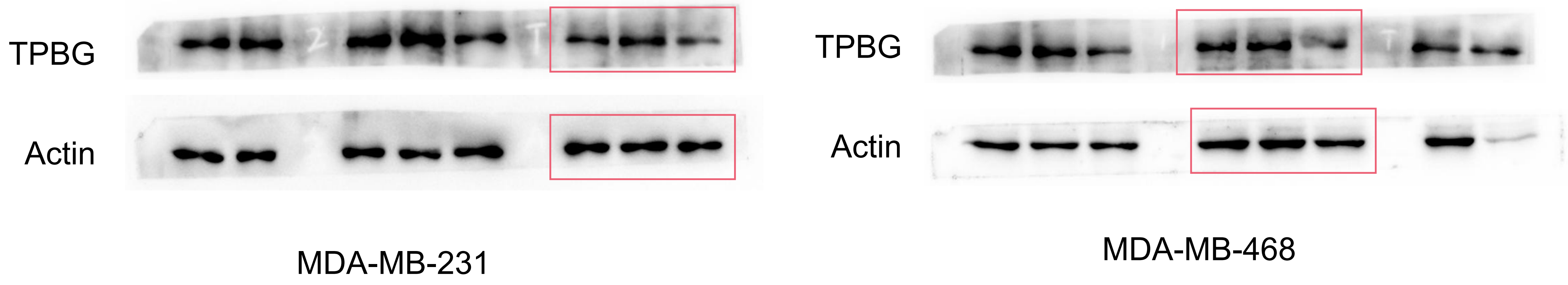

TPBG
Actin
MDA-MB-231
TPBG
Actin
MDA-MB-468

Fig. 8E

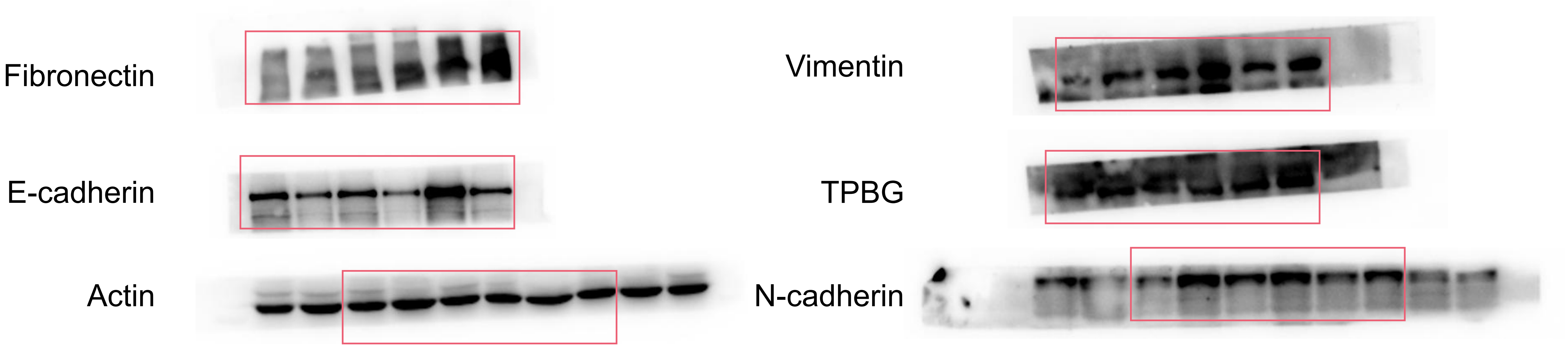

Fibronectin
E-cadherin
Actin
Vimentin
TPBG
N-cadherin

Fig. S1B

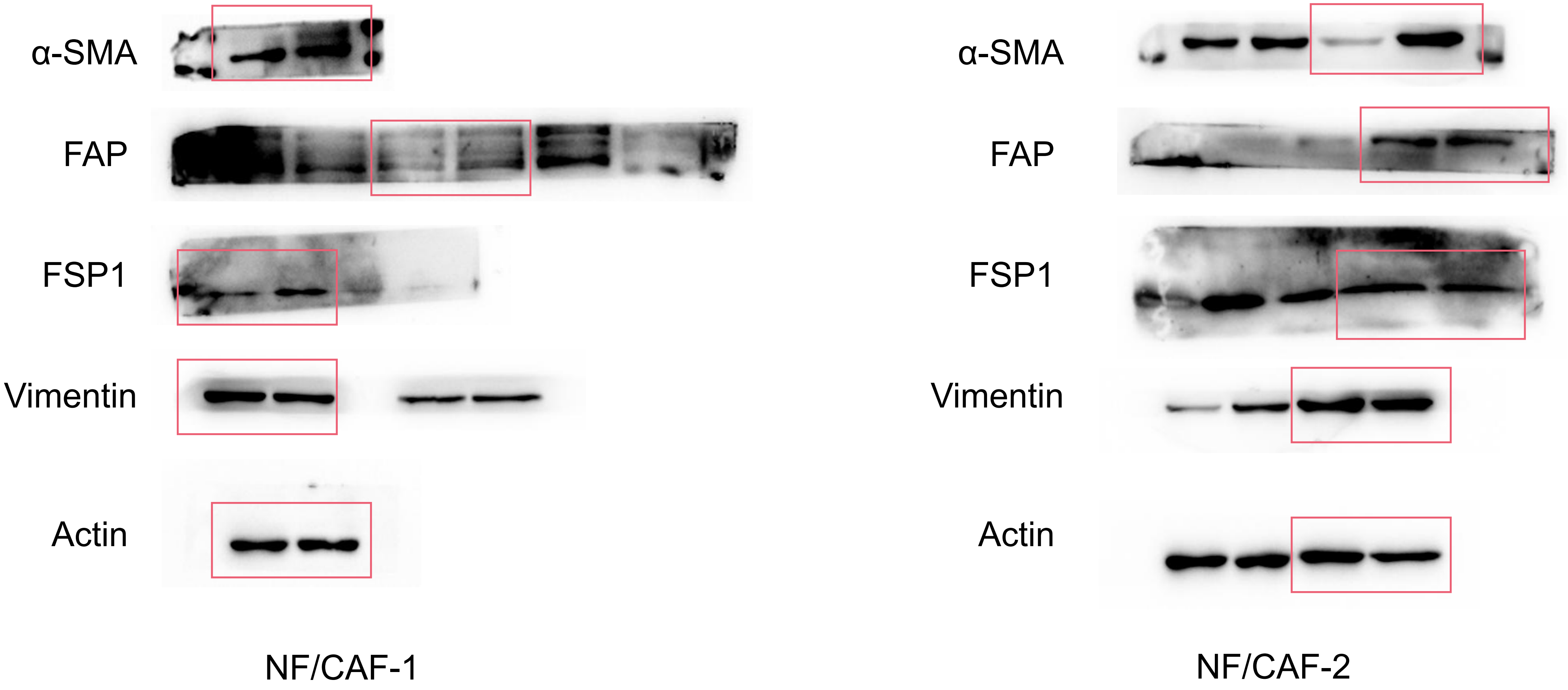
α-SMA
FAP
FSP1
Vimentin
Actin
NF/CAF-1
α-SMA
FAP
FSP1
Vimentin
Actin
NF/CAF-2

Fig. S2A

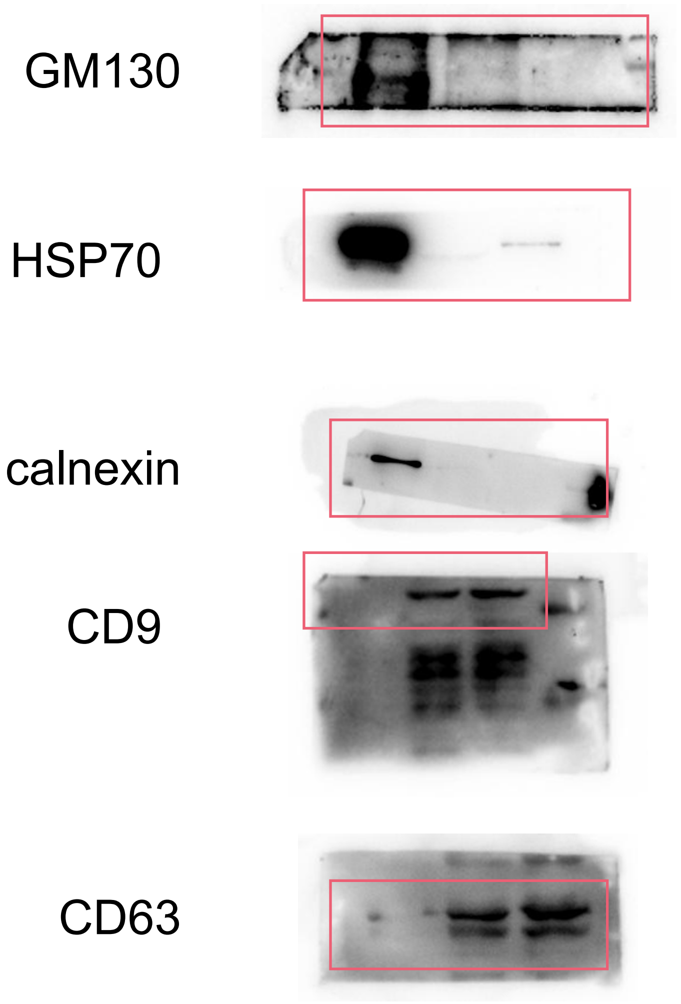

Fig. S4G

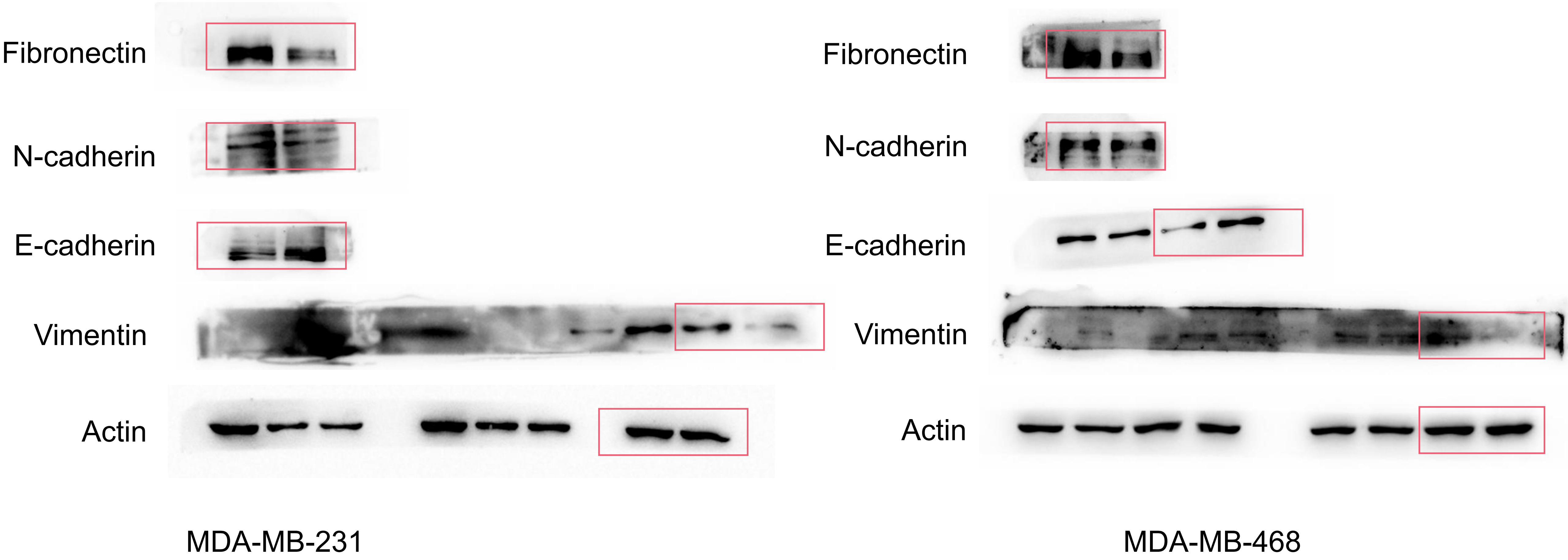
Fibronectin
N-cadherin
E-cadherin
Vimentin
Actin
MDA-MB-231
Fibronectin
N-cadherin
E-cadherin
Vimentin
Actin
MDA-MB-468

Fig. S8A

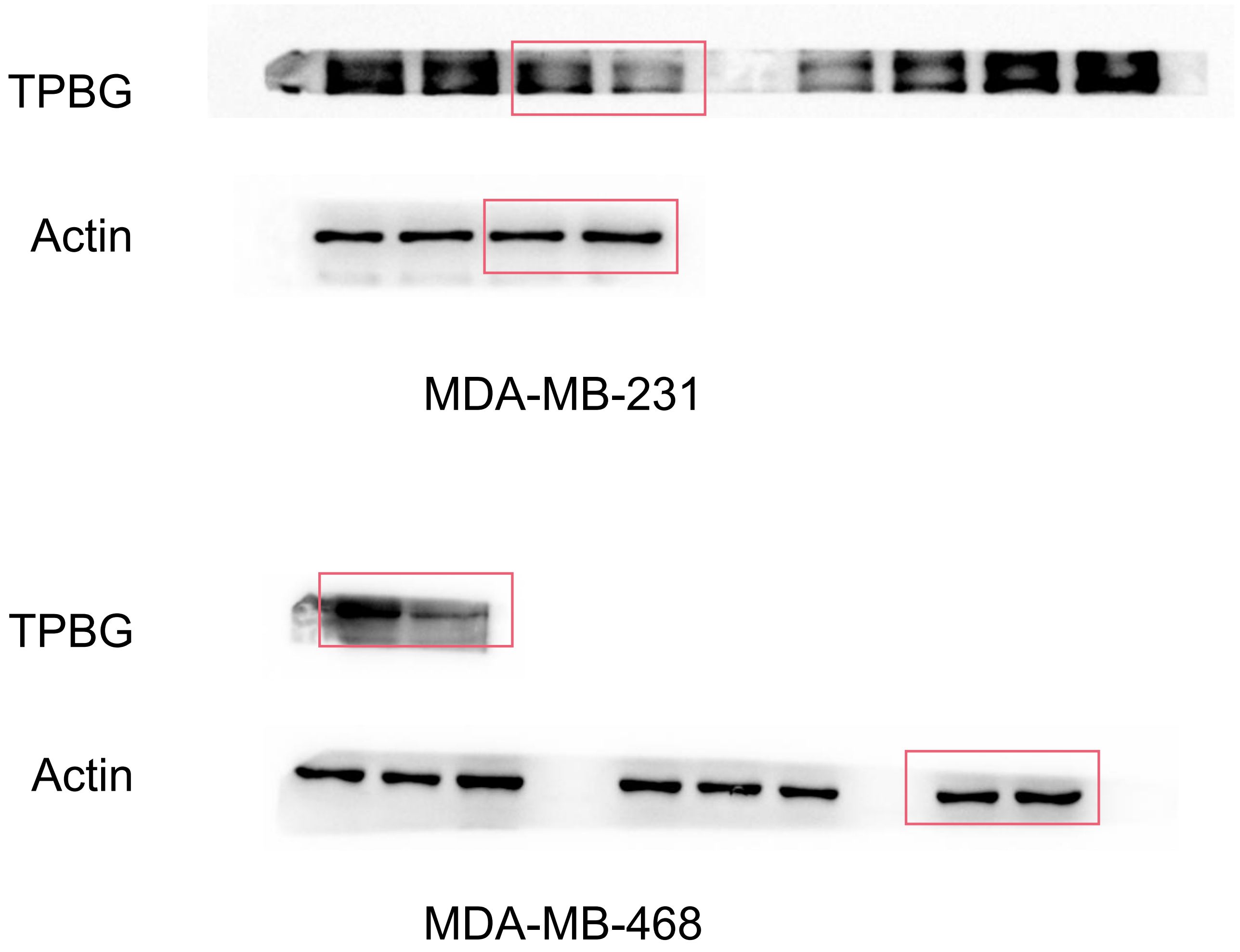

Supplement: Supplementary file 14 — Original Data File [file 41419_2023_5986_MOESM14_ESM.pdf]
